# Using RNA-seq to Profile Gene Expression of Spikelet Development in Response to Temperature and Nitrogen during Meiosis in Rice (*Oryza sativa* L.)

**DOI:** 10.1371/journal.pone.0145532

**Published:** 2015-12-29

**Authors:** Jun Yang, Xiaorong Chen, Changlan Zhu, Xiaosong Peng, Xiaopeng He, Junru Fu, Linjuan Ouyang, Jianmin Bian, Lifang Hu, Xiaotang Sun, Jie Xu, Haohua He

**Affiliations:** 1 Key Laboratory of Crop Physiology, Ecology and Genetic Breeding, Ministry of Education, College of Agronomy, Jiangxi Agricultural University, Nanchang, 330045, China; 2 Southern Regional Collaborative Innovation Center for Grain and Oil Crops, Hunan Agricultural University, Changsha, 410128, China; Jawaharlal Nehru University, INDIA

## Abstract

Rice reproductive development is sensitive to high temperature and soil nitrogen supply, both of which are predicted to be increased threats to rice crop yield. Rice spikelet development is a critical process that determines yield, yet little is known about the transcriptional regulation of rice spikelet development in response to the combination of heat stress and low nitrogen availability. Here, we profiled gene expression of rice spikelet development during meiosis under heat stress and different nitrogen levels using RNA-seq. We subjected plants to four treatments: 1) NN: normal nitrogen level (165 kg ha^-1^) with normal temperature (30°C); 2) HH: high nitrogen level (264 kg ha^-1^) with high temperature (37°C); 3) NH: normal nitrogen level and high temperature; and 4) HN: high nitrogen level and normal temperature. The *de novo* transcriptome assembly resulted in 52,250,482 clean reads aligned with 76,103 unigenes, which were then used to compare differentially expressed genes (DEGs) in the different treatments. Comparing gene expression in samples with the same nitrogen levels but different temperatures, we identified 70 temperature-responsive DEGs in normal nitrogen levels (NN vs NH) and 135 DEGs in high nitrogen levels (HN vs HH), with 27 overlapping DEGs. We identified 17 and seven nitrogen-responsive DEGs by comparing changes in nitrogen levels in lower temperature (NN vs HN) and higher temperature (NH vs HH), with one common DEG. The temperature-responsive genes were principally associated with cytochrome, heat shock protein, peroxidase, and ubiquitin, while the nitrogen-responsive genes were mainly involved in glutamine synthetase, amino acid transporter, pollen development, and plant hormone. Rice spikelet fertility was significantly reduced under high temperature, but less reduced under high-nitrogen treatment. In the high temperature treatments, we observed downregulation of genes involved in spikelet development, such as pollen tube growth, pollen maturation, especially sporopollenin biosynthetic process, and pollen exine formation. Moreover, we observed higher expression levels of the co-expressed DEGs in HN vs HH compared to NN vs NH. These included the six downregulated genes (one pollen maturation and five pollen exine formation genes), as well as the four upregulated DEGs in response to heat. This suggests that high-nitrogen treatment may enhance the gene expression levels to mitigate aspects of heat-stress. The spikelet genes identified in this study may play important roles in response to the combined effects of high temperature and high nitrogen, and may serve as candidates for crop improvement.

## Introduction

More than half of the world’s population relies on rice (*Oryza sativa* L.) as a staple food. The human population is expected to increase by 50% by the end of the century, creating to an elevated need for rice production meet the dietary needs of the global population [1]. There was a boost in rice production over the last decades through the elevated application of nitrogen fertilizers. The global use of nitrogen fertilizers augments plant productivity, as a consequence of the fact that most of the high-yield rice varieties have high demands of nitrogen. However, decreasing levels of soil nitrogen caused by increased farming continues to be a challenge that influences rice yield. Another challenge is the increasing global temperatures caused by increased atmospheric CO_2_ concentration. Heat can drastically reduce rice yield, and it is projected that rice will be more frequently subjected to high temperature [2,3,4]. Taken together, there is a need to better understand how nitrogen and temperature affect rice growth and development.

Heat stress has strong negative effects on rice plant development. Exposing rice during the reproductive stage, especially during meiosis to temperatures higher than 35°C leads to reduced spikelet fertility, which is caused by poor anther dehiscence, low pollen production, and low numbers of germinating pollen grains on the stigma of rice [5,6,7]; this in turn drastically reduces yield [8,9]. Thus spikelet fertility under high temperature can be regarded as a trait to screen for heat tolerance during the reproductive stage [7,8].

Accompanying these phenotypic changes, rice plants also alter gene expression in response to heat stress. Using a rice microarray [10], the gene expression profile of rice panicle grown at 40°C during anther development shows changes in expression of genes involved in transcriptional regulation, transport, cellular homeostasis, and stress response. The predominant transcription factor families that respond to heat stress are Hsf, NAC, AP2/ERF, WRKY, MYB, and C_2_H_2_ [10]. In the anther, there have been 13 genes identified as repressed in high temperatures. These 13 genes are mostly expressed in the immature anther’s tapetum during the microspore stage, and become downregulated after being exposed to high temperature [6]. Interestingly, the expression levels of tapetum-specific genes, including *Osc6*, *OsRAFTIN* and *TDR*, are not affected by high temperature [6]. *Indica* rice N22 is a heat-tolerant rice variant with 71% spikelet fertility at 38°C, and specifically shows high heat tolerance during anthesis. Compared to the non-heat resistant varieties, N22 shows significant upregulation of cold and heat shock proteins, which may contribute to the heat tolerance of N22 [7]. Nevertheless, the detailed molecular mechanism of heat response in rice spikelet during meiosis remains unclear.

Nitrogen is the most important inorganic nutrient for plant growth and development, and its deficiency causes significant loss of rice yield [11]. Nitrogen is incorporated into the signaling pathways that regulate plant growth and development [12,13,14,15]. Grain yield is correlated with soil nitrogen availability between above 150 to 219 kg N ha^-1^ [11]. The effect of nitrogen limitation is best understood in *Arapidopsis*, where one study found that plants show altered expression of 629 genes in response to low nitrogen levels. About half of these genes were upregulated, and these genes included those involved in protein degradation and the biosynthesis of anthocyanin. The other half were downregulated, and included genes involved in photosynthesis and the synthesis of nitrogenous macromolecules such as chlorophyll, proteins, amino acids and nucleotides [16]. In seedlings exposed to low nitrogen, Arabidopsis showed difference in about 70 genes, while rice plants showed differential expression of 10,422 genes [17] 25 and 49 nitrogen-responsive genes to low or high nitrate induction were identified in *Arabidopsis* seedlings, respectively [18]. Nitrogen responsive genes encode transcription factors, proteins involved in signal transduction process, and hormone synthesis and response [19,20,21]. However, specific mechanism of how these genes and pathways respond to nitrogen use is still unclear.

Given that nitrogen levels and heat stress are both important conditions that regulate rice plant growth, some studies have investigated the combined effect of nitrogen and temperature. In rice, the photosynthetic rates and respiratory rate are correlated with the leaf nitrogen content and temperature [22]. Temperature and nitrogen levels can influence shoot nitrogen concentrations, where the concentrations are higher in plants subjected to high night-time temperature while being exposed to 40 or 160 mg N L^-1^, but this increase is not observed in plants grown in very low nitrogen levels [23]. In addition, leaf area, plant height, root maximum length, root and shoot nitrogen concentrations, soluble leaf protein content, and soluble leaf carbohydrate content are greater in plants treated with high night-time temperature when plants are exposed to 40 or 160 mg N L^-1^ [23]. High nitrogen levels during spikelet differentiation stage to the young microspore stage greatly increases the sensitivity to low temperature [24]. In addition, cool temperature (12°C) treatment for three days decreases rice spikelet fertility by 36% under standard-nitrogen and 42% under high-nitrogen conditions [25]. These findings highlight the complex relationship of nitrogen levels and temperature, suggesting that there may be an optimal combination of temperature and nitrogen levels for each plant/variant, a combination that may be modifiable through genetic engineering. As spikelet fertility is a key indicator of rice yield, a better understanding of how nitrogen interacts with high temperature can help with determining the processes/genes that are relevant to increase rice yield.

Over the last decade, RNA-seq has provided a high-throughput way to identify the DEGs [26], and to generate transcriptional profiling that is more accurate compared to microarray studies [27,28]. Rice is a gramineous monocot model with a well-sequenced genome, which provides a good reference for generating de novo transcriptomes. Here, in order to increase our understanding of the molecular mechanisms of rice spikelet development, we conducted RNA-seq and digital gene expression profile (DGE) analysis to identify candidate genes involved in the response to high temperature and high nitrogen levels in rice spikelet that are undergoing meiosis. We further tested whether the two conditions interact with each other.

## Materials and Methods

### Plant culture and nitrogen treatment

Ganxin203, a super-hybrid early rice (*Oryza sativa* L. ssp. *indica*) variety, was grown in hydroponic conditions in 2014 at High-Tech Agricultural Science and Technology Park of Jiangxi Agricultural University (latitude: 28° 46′ N, longitude: 115° 50′ E, altitude: 48.80m), Jiangxi Province, China. Seed dormancy was broken by incubating seeds at 50°C for 3 days, followed by pre-germination, then sown in a rice field. 30 day old seedlings were transplanted on 23 April 2014 at a spacing of 22.5cm × 24.5cm with one seedling per hill. The soil was clay type with pH 5.94, 28.72 g kg^-1^ organic matter, 1.45 g kg^-1^ total nitrogen, 92.01 mg kg^-1^ available nitrogen, 28.31 mg kg^-1^ available phosphorous (P), and 221.67 mg kg^-1^ available potassium (K). The soil properties were based on samples taken from the top 20cm of the soil. Plants were subjected to one of four treatments composed of two factors, nitrogen and temperature: 1) NN: normal nitrogen level (165 kg ha^-1^, as the control) with normal temperature (30°C, as the control); 2) HH: high nitrogen level (264 kg ha^-1^) with high temperature (37°C); 3) NH: normal nitrogen level and high temperature; and 4) HN: high nitrogen level and normal temperature. Plants subjected to the normal nitrogen level treatment were applied with 66 kg N ha^-1^ (as pure N) at basal (2 d before transplanting), 33 kg N ha^-1^ at tillering (10 days after transplanting), and 66 kg N ha^-1^ at panicle initiation (30 days after transplanting). Plants under high nitrogen level were treated with 66, 66, and 132 kg N ha^-1^ at basal, tillering, and panicle initiation, respectively [29]. For all conditions, phosphorus fertilizer was applied at 90 kg P_2_O_5_ ha^-1^ 2 days before transplanting, and potassium fertilizer was applied at 180 kg K_2_O ha^-1^ in two equal splits at basal and panicle initiation. Standard cultural practices were used for crop management, weed in the field was manually removed, and all insects were rigorously controlled through chemical insecticides. The experimental field remained flooded from the day of transplanting until 10 days before maturity.

### High temperature treatment

Temperature of the plants was controlled in four temperature-controlled growth cabinets at high temperature (37°C) and normal temperature (30°C). Each cabinet (PRX–1500B, internal size: length 1.91m × width 0.76m × height 1.83m, Shanghai Bilon Instruments Co., Ltd., China) was fixed at a 5m interval to ensure adequate ventilation. For high temperature analysis, rice plants, along with the surrounding soil, at the female stamen primordium differentiation stage were randomly transplanted into plastic pots to adapt for 3 days, and then placed in the growth cabinets. The pot had a hole at the bottom with an internal diameter of 17cm and height of 16cm, and the rice plants that were transferred into the pots grew without withering and yellow leaves. Twelve rice plants at the formation stage of pollen mother cell [6] were randomly subjected to high temperature (from 8:00 h to 18:00 h, Beijing time, 37°C; from 18:00 h to the next morning 8:00 h, 30°C), or normal temperature (from 8:00 h to 18:00 h, 30°C; from 18:00 h to the next morning 8:00 h, 25°C) for 4 d [7,9,30]. The temperature was monitored every 15 mins using two standalone sensors that were placed in the rice canopy near the panicle base (few centimeters into the canopy) in each cabinet, and all the sensors were connected to data loggers (HOBO, U22–001, USA) [31,32]. All other controlled environmental conditions, such as white fluorescent illumination of 540 μmol m^-2^ s^-1^ day/ 0 μmol m^-2^ s^-1^ night, and relative humidity of 75% day/ 80% night, were consistent for each growth cabinet. The treatments were repeated twice, and each repeat had six plants (pots) per growth cabinet. Young florets were collected in the same phases of meiosis, as described by Endo et al [6] and Tang et al [33]. Features used to distinguish this stage were: a) rice was grown for about 2 months, and were 13 to 19 days before flowering; b) the distance between the auricle of the flag leaf and the penultimate leaf was within 1cm; c) the young panicle length was about 2cm; d) the floret length was 2 to 3mm. 4 days after high temperature treatment, young florets undergoing meiosis of the pollen mother cell during middle of main panicles were collected, frozen in liquid nitrogen, and stored at -80°C. For each treatment, two replicates were collected, with each replicate containing samples from 3 plants.

After plants were subjected to different temperature treatments, the pots were removed and the remaining 6 rice plants were transplanted back to natural field conditions. The average temperatures in the each cabinets were similar to chamber conditions: high temperature (from 8:00 h to 18:00 h, actual, 36.58°C, SE = ±0.13°C; from 18:00 h to the next morning 8:00 h, 29.65±0.05°C), and normal temperature (from 8:00 h to 18:00 h, 29.14±0.09°C; from 18:00 h to the next morning 8:00 h, 25.88±0.07°C).

### Quantification of yield and spikelet fertility (seed-set)

Rice plants were sampled from each plot when plants reached physiological maturity. Spikelet fertility (seed-set) was estimated according to Mohammed et al [32]. Four plants were randomly selected, and two main tiller panicles per plant (eight total) were tagged and harvested in each treatment. Spikelet fertility was determined using the ratio of the number of filled grains to the total number of reproductive sites (florets). Whether the grain was filled or not was determined by pressing each floret between the forefinger and thumb. Grains were considered filled for both completely and partially filled grains. The spikelet fertility data were statistically analyzed using the analysis of variance (ANOVA; SPSS version 17.0), and the mean separation was performed using the least significant difference (LSD) at 5% probability.

### Transcriptome library creation and sequencing

Total RNA from young florets undergoing meiosis was isolated using TRIzol reagent (Invitrogen) according to the manufacturer’s protocol. For transcriptome sequencing and assembly, RNA from all four treatments were mixed and pooled equally to obtain more sequence information, however, each treatment was subjected individually to digital gene expression (DGE) sequencing. Oligo(dT) beads were used to isolate poly(A) + mRNA from total RNA, and mRNA were disrupted into short fragments using fragmentation buffer. These short fragments were used as templates for random hexamer primer to synthesize first-strand cDNA. The second-strand cDNA was synthesized by adding buffer, dNTPs, RNase, and DNA polymerase I. The resulting short fragments were purified with a PCR extraction kit and resolved with EB buffer for end reparation and addition of a poly(A) tail. Next, the short fragments were connected with sequencing adapters. Following agarose gel electrophoresis, suitable fragments were selected as templates for PCR. The library was sequenced using an Illumina HiSeq^TM^ 2000 platform, performed at the Beijing Genomics Institute (BGI, http://www.genomics.cn/index; Shenzhen, China) (S1 Fig). The raw reads were stored in a fastq format.

### *De novo* transcriptome assembly

We found some adaptor sequences and/or low quality reads in the raw reads, therefore reads were filtered into high quality clean reads (or clean data) using data cleaning (or data filtering). Reads containing adapter sequences, low-quality sequences (reads with ambiguous bases ‘N’) and reads with more than 10% in Q20 bases were removed, then the clean reads were used for *de novo* assembly.

Assembly of the transcriptome was conducted using the short reads assembly program Trinity [34]. Trinity combines three independent software modules: Inchworm, Chrysalis, and Butterfly, applied sequentially to process large volumes of RNA-seq reads. Contigs were assembled using reads containing a certain length of overlap and no uncalled bases (N). Scaffolds were assembled by connecting contigs using N to represent the unknown sequence between each pair of contigs. Paired-end reads were used for gap filling of scaffolds to obtain sequences with the smallest number of Ns, which were then defined as unigenes. To ensure that the unigenes were non-redundant, the assembly was further processed for sequence splicing and redundancy removing. Once redundancy was removed, the gene family clustering was conducted by dividing unigenes into two classes: clusters or singletons. Clusters were assigned the prefix CL followed by the cluster ID. Thesame cluster contained several unigenes with similarity of more than 70%. Singletons were assigned the prefix of the unigene.

Lastly, the unigenes were searched against protein databases including the National Center for Biotechnology Information (NCBI) non-redundant (NR) database, Swiss-Prot (A manually annotated and reviewed protein sequence database), Kyoto Encyclopedia of Genes and Genomes (KEGG; http://www.genome.jp/kegg/) and Clusters of Orthologous Groups (COG). These were searched using BLASTX with E-value < 10^−5^ as a cutoff, and the best-hits were used to decide the sequence direction for each unigene. If the search results of different databases conflicted with each other, a priority was given by the order of NR, Swiss-Prot, KEGG, and COG. If a unigene did not align to any of the databases, the software ESTScan [35] was used to determine the sequence direction. For unigenes with assigned sequence directionsthe sequences were listed from 5' to 3' end; for those without information on transcript direction, we provided their sequences that were obtained from the software assembly (S2 Fig).

### Statistics of digital gene expression (DGE) sequencing

After DGE sequencing, clean reads were mapped to the above-generated reference genes (*de novo* assembly) and/or reference genome (*Indica* rice database) using SOAPaligner/ SOAP2 [36]. No more than two mismatches were allowed in each alignment. Randomness was evaluated using the distribution of reads on the reference genes [28]. Genes with similar expression patterns usually indicated functional correlation. We performed a cluster analysis of gene expression patterns with cluster software [37] and Java Treeview software [38]. Other evaluations included quality assessment of reads, sequencing saturation analysis, gene sequencing coverage, and correlation analysis of all genes between every two samples replicates.

### Functional annotation and classification

We functionally annotated the unigene sequences by first aligning sequences using BLASTX to the NR, Swiss-Prot, KEGG and COG protein databases (E-value < 10^−5^), then using BLASTN to nucleotide database NT (e-value<0.00001). This allowed for retrieving proteins with the highest sequence similarity to unigenes along with their protein functional annotations (S3 Fig).

After NR annotation, the distribution of gene functions at the macro level were annotated using the Blast2GO program [39] to obtain Gene Ontology (GO) annotations, and WEGO software [40] to perform GO functional classification of all unigenes. GO is a standardized gene functional classification system, and covers three domains: cellular component, molecular function and biological process. GO enrichment analysis allows for comparing the biological functions of differentially expressed genes (DEGs). The DEGs were mapped to the GO terms in the database (http://www.geneontology.org/), calculating gene numbers for every term. Significantly enriched GO terms were then determined using hypergeometric test. The calculating formula is:

$$P=1-\overset{m-1}{\underset{i=0}{\sum }}\frac{\left(\begin{array}{l} M\\ i \end{array}\right)\left(\begin{array}{l} N-M\\ n-i \end{array}\right)}{\left(\begin{array}{l} N\\ n \end{array}\right)}$$

Where N is the number of all genes with GO annotation; n is the number of DEGs in N; M is the number of all genes that are annotated to the certain GO terms; m is the number of DEGs in M. The calculated P-value were analyzed through Bonferroni Correction, taking corrected P-value < = 0.05 as a threshold. GO terms fulfilling this condition ere defined as significantly enriched GO terms in DEGs.

KEGG is a public pathway-related database [41,42]. Pathway enrichment analysis identifies significantly enriched metabolic pathways or signal transduction pathways in DEGs. The calculating formula was as the same as that in GO analysis.

After having obtained the GO and KEGG annotation for all unigenes, unigenes were then aligned to the COG database to predict and classify potential functions based on known orthologous gene products. The COG database takes into consideration the shared evolutionary origin of proteins, and the whole database is built on coding proteins with complete genomes from bacteria, algae and eukaryotic organisms [43].

### Identification of differentially expressed genes (DEGs)

Unigene expression was calculated using the reads per kb per million reads method (RPKM) [44], with the following formula:

$$RPKM=\frac{10^{6}C}{NL/10^{3}}$$

Where RPKM (A) is the expression of unigene A, and C is the number of reads that uniquely aligned to unigene A. N is the total number of reads that uniquely aligned to all unigenes, and L is the base number of unigene A. The RPKM method eliminates the influence of gene length and sequencing level on the calculation of gene expression [44].

To compare the differences in gene expression, the read frequency in each DGE library was statistically analyzed according to the method of Audic and Claverie [45]. The false discovery rate (FDR) was used to determine the threshold P-value in multiple tests. A FDR below 0.001 and an absolute E-value of the log2 ratio above one were used as the threshold to determine significant differences in gene expression [46]. Following these tests, DEGs were subjected to GO and KEGG Ontology (KO) enrichment analysis (S4 Fig).

### DEGs validation by real-time quantitative PCR (RT-qPCR)

RT-qPCR was performed at the Beijing Genomics Institute (BGI; Shenzhen, China). Total RNA was extracted using the same method as the DEG library preparation. Total RNA (2 μg) from each sample was reverse-transcribed in a 10.5 μL reaction. First-strand cDNA synthesis was conducted using random hexamer primers and reverse transcriptase (Invitrogen). A total of 10 randomly selected genes were analyzed by RT-qPCR using the SYBR Green PCR Mix (QIAGEN) and the ABI ViiA 7 real-time PCR system (Applied Biosystems). The primer sequences are listed in S1 Table. The 16S was used as endogenous control in RT-qPCR analysis. The PCR-cycling conditions were: 95°C for 2 min, followed by 40 cycles at 94°C for 10 s, 58°C for 10 s and 72°C for 40 s. After each PCR run, the melting curve was analyzed to ensure the specificity of the product. Calculation of relative quantification was done by the comparative 2^-ΔΔCt^ method [47]. All reactions were performed in triplicates per sample.

## Results

### RNA sequencing and *de novo* assembly of transcriptome

To understand the gene expression changes of rice spikelet in response to the combined stress of heat and nitrogen, we first generated a *de novo* transcriptome. A pooled RNA sample of rice spikelets during meiosis from plants that were subjected to various combination of heat and nitrogen samples were sequenced using Illumina second generation high-throughput sequencing.

**Fig 1:**
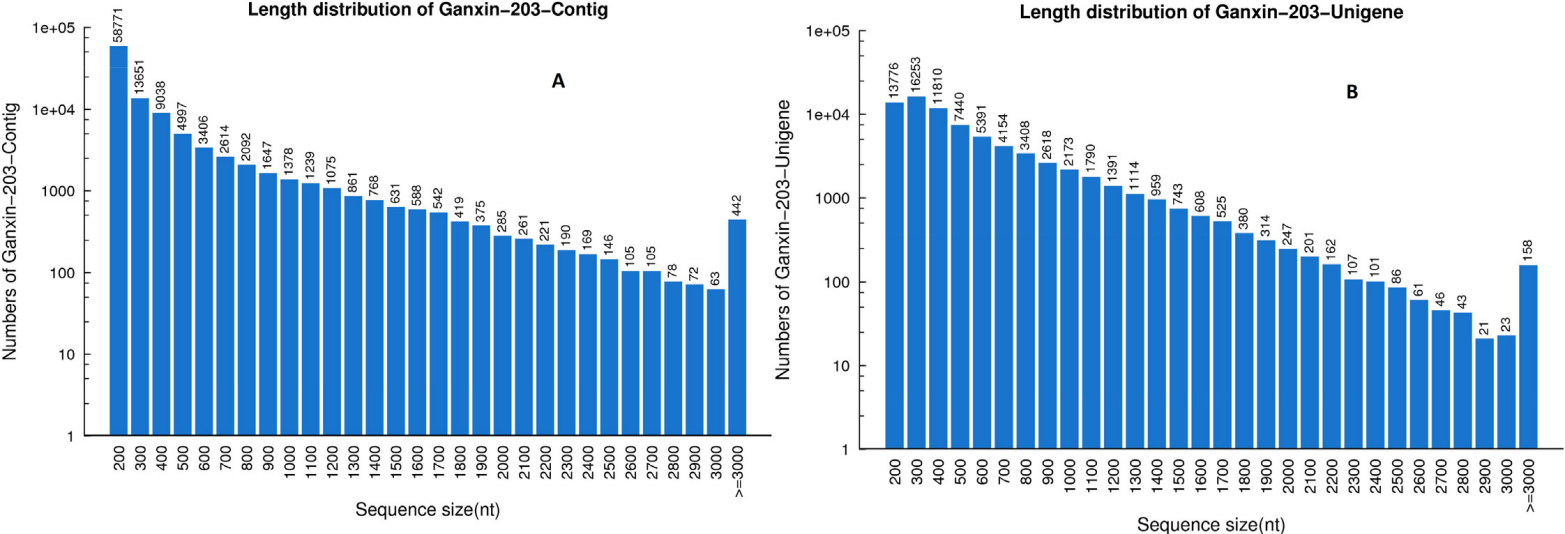
The length distribution of the *de novo* assembly for contigs (A) and unigenes (B). The horizontal coordinates are contig lengths (A) or unigenes lengths (B), and the vertical coordinates are numbers of contigs (A) or unigenes (B). The length distribution of contigs and unigenes were counted from 200 nt to 3000 nt with an interval of 100 nt. Each number in the x-axis indicates a region of sequence length covering 100 nt, for example, ‘‘200” represents a region of sequence length between 200 to 300.

We obtained a total of 52,250,482 clean reads (accumulated nucleotides, 4,702,543,380 bp), which were assembled into 106,229 contigs with Q20 percentage and GC content of 96.32%, and 52.98%, respectively. We further assembled the contigs into 76,103 unigenes, with a mean length of 520 bp (Table 1). The contig and unigene size distributions suggest that the Illumina sequencing was reproducible and reliable (Fig 1A and 1B).

**Table 1:** Summary of transcriptome sequencing.

Total raw reads	60,889,348
Total clean reads	52,250,482
Total clean nucleotides (bp)	4,702,543,380
Q20 percentage	96.32%
GC percentage	52.98%
Total number of contigs	106,229
Total length of contigs (bp)	38,658,990
Mean length of contigs (bp)	364
N50 of contigs (bp)	656
Total number of unigenes (bp)	76,103
Total length of unigenes (bp)	39,546,458
Mean length of unigenes (bp)	520
N50 of unigenes (bp)	712

### Functional annotation and classification of the transcriptome

We annotated the transcriptome by blasting all the distinct unigene sequences against NR, NT, Swiss-Prot, KEGG, COG, and GO databases by BLASTX with a cut-off E-value of 10^−5^. This resulted in a total of 75,807 unigenes (99.61% of all unigenes) that were above the cut-off value (Table 2). 60,788 unigenes were annotated by NR (79.88% of all unigenes; Table 2), and 75,593 (99.33%), 34,776 (45.70%), 31,311 (41.14%), 18,041 (23.71%), and 44,131 (57.99%) unigenes were annotated by NT, Swiss-Prot, KEGG, COG, and GO databases, respectively.

**Table 2:** Statistics of unigenes annotation using public databases.

Item	NR	NT	Swiss-Prot	KEGG	COG	GO	ALL
Total number of annotated unigenes	60,788	75,593	34,776	31,311	18,041	44,131	75,807
Percentage of annotated unigenes (%)	79.88	99.33	45.70	41.14	23.71	57.99	99.61

For unigenes annotated using the NR database, we found 25,492 (41.94%) unigenes with E-value lower than 1.0e-60, and 35,296 (58.06%) unigenes with E-values between 1.0e-60 to 1.0e-5 (S5A Fig). Most unigenes (54,882; 90.29%) had sequence similarity between 80–100%, and. only 5,906 (9.71%) of unigenes had less than 80% sequence similarity (S5B Fig). We found that 36,498 (60.04%) unigenes homology to rice (*Oryza sativa* L. ssp. *japonica*) sequences. Interestingly, only 21,215 (34.90%) unigenes had strong homology to rice (*Oryza sativa* L. ssp. *indica*) sequences (S5C Fig).

**Fig 2:**
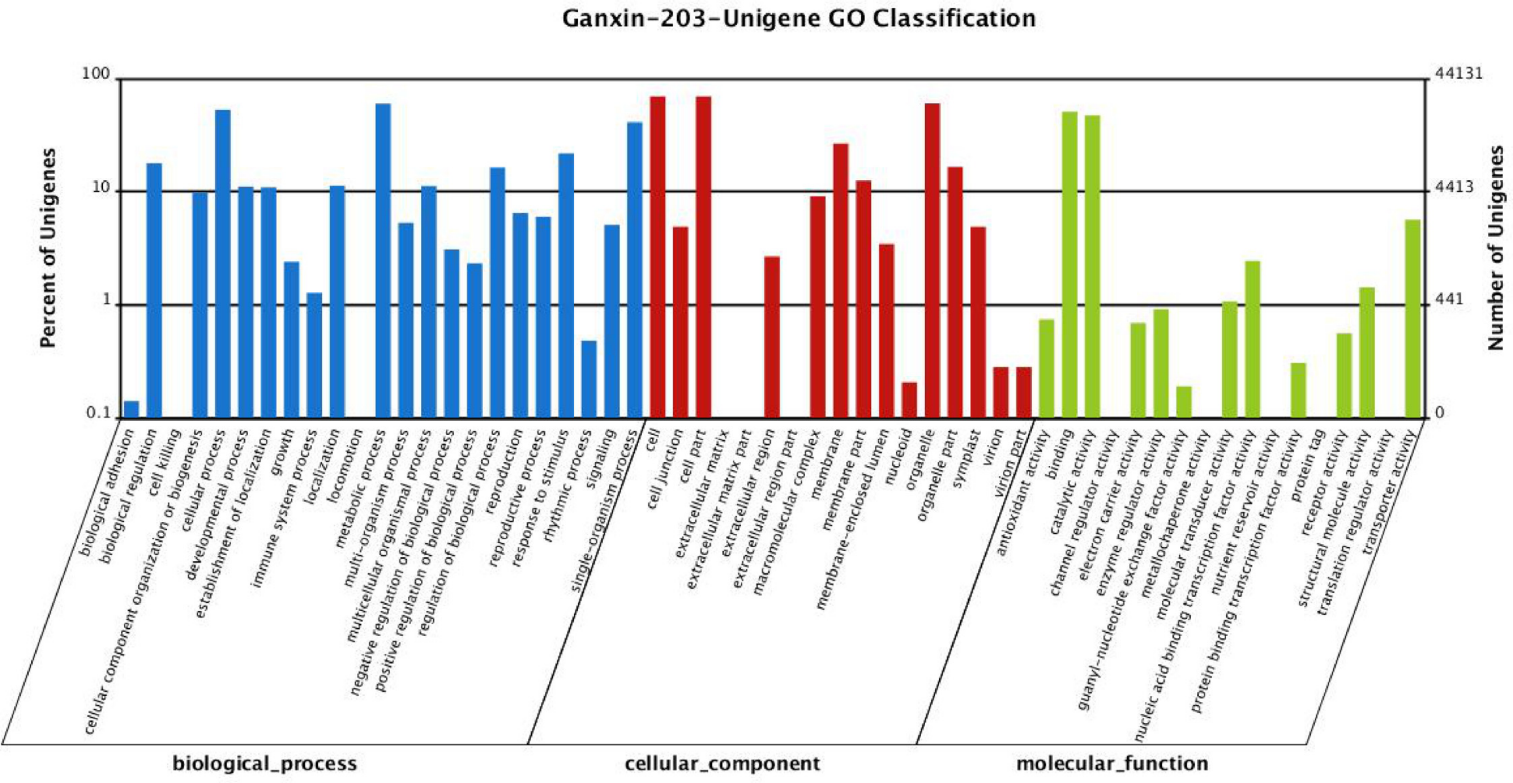
GO analysis of unigenes. GO functions are listed in the X-axis. The Y-axis represents the number (right) and percentage (left) of genes that have the GO function.

We classified the functions of the predicted genes using Gene Ontology (GO) assignments. Based on sequence homology, 44,131 unigenes and 304,589 sequences,were categorized into 57 functional groups (Fig 2). In each of the three main categories (biological process, cellular component, and molecular function) of the GO classification, the major subcategories were: “metabolic process”, “cellular process”, and “single-organism process” for biological process, “cell”, “cell part”, and “organelle” for cellular components, and “binding”, “catalytic activity”, and “transporter activity” for molecular function.

**Fig 3:**
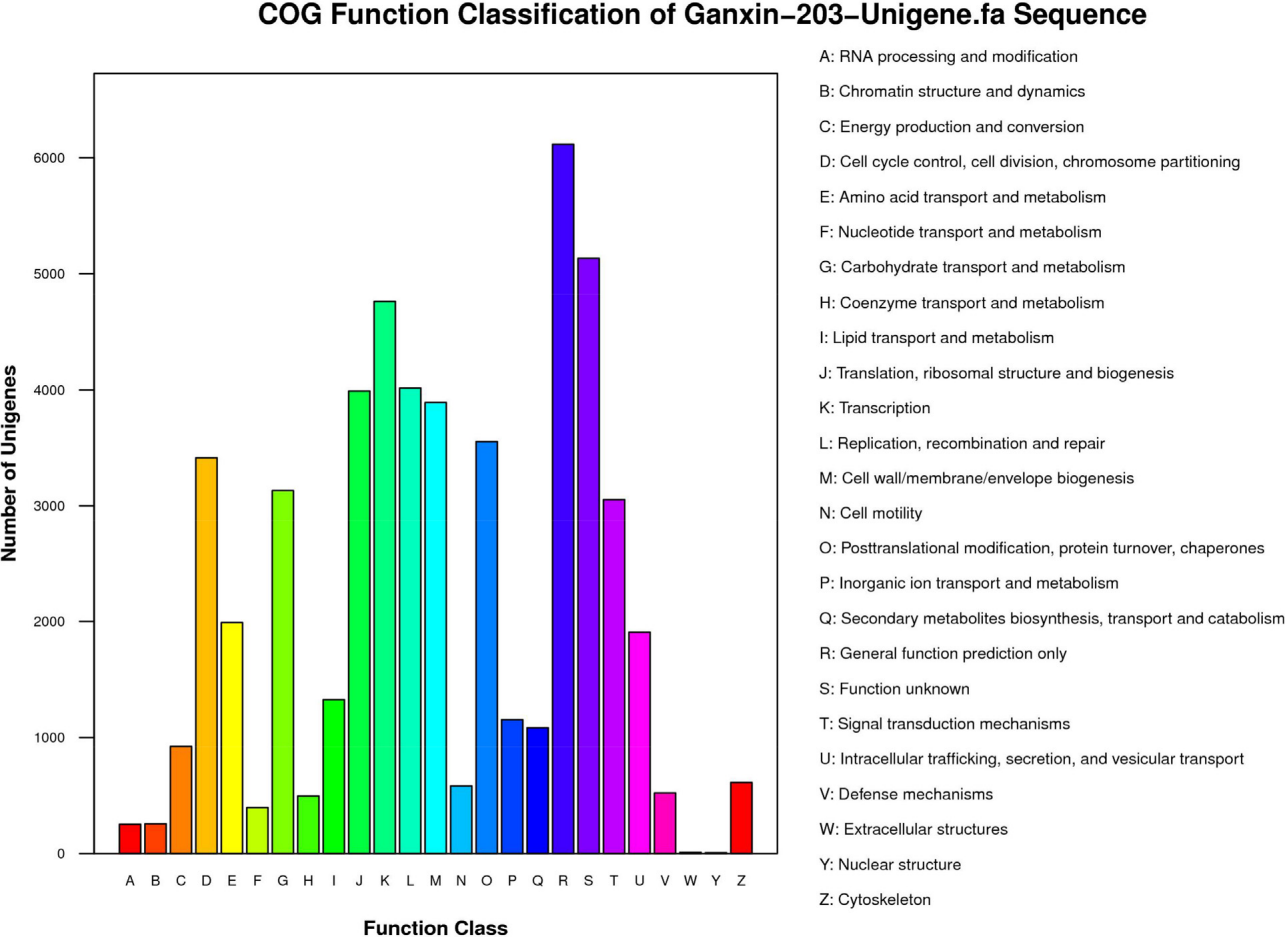
COG function classification of unigenes.

The sequences were further evaluated using the COG and KEGG databases. Out of the 18,041 annotated unigenes, 52,582 unigenes were categorized into 25 COG categories (Fig 3). Among them, the largest group fell under the cluster ‘‘general function prediction only (6,116)”, “function unknown” (5,133), “transcription” (4,766), “replication, recombination, and repair” (4,017), and “translation, ribosomal structure and biogenesis” (3,992). We conducted functional classification and pathway assignment using the KEGG database, and assigned a total of 31,311 unigenes to 128 KEGG pathways. The top ten pathways are listed in Table 3.

**Table 3:** The top 10 KEGG pathway classifications of the transcriptome. The small brackets denote the percentage of annotated unigenes in a pathway to the total number of unigenes.

Number	Pathway	Unigenes with pathway annotation (31,311)	Pathway ID
1	Metabolic pathways	8,697 (27.78%)	ko01100
2	RNA transport	3,774 (12.05%)	ko03013
3	mRNA surveillance pathway	3,289 (10.5%)	ko03015
4	Endocytosis	3,103 (9.91%)	ko04144
5	Glycerophospholipid metabolism	3,070 (9.8%)	ko00564
6	Biosynthesis of secondary metabolites	3,014 (9.63%)	ko01110
7	Ether lipid metabolism	2,872 (9.17%)	ko00565
8	Plant-pathogen interaction	1,673 (5.34%)	ko04626
9	Spliceosome	1,458 (4.66%)	ko03040
10	Plant hormone signal transduction	1,343 (4.29%)	ko04075

### Digital gene expression (DGE) library sequencing

To investigate the differences in gene expression profiles of spikelets in rice subjected to different heat/nitrogen treatments, we constructed and sequenced four DGE libraries from the following treatment conditions: normal nitrogen levels with high temperatures (NH), normal nitrogen and temperature levels (NN), high nitrogen and temperature levels (HH), and high nitrogen levels with normal temperature (HN). Sequences were first tested by Agilent 2100 for quality control (S2 Table), and each library contained approximately 11 to 13 million high-quality reads (Table 4). We mapped the sequences of the DGE libraries to our transcriptome reference database. We found similar results by mapping reads to the reference genes (*de novo* assembly of transcriptome) (Table 4) and to the reference genome (*Indica* rice database) (S3 Table). The percentage of clean reads relative to the raw reads in each library was above 98% (S6 Fig). 9 to 11 million clean reads were mapped to unigenes, and we recovered more than 82% of clean reads in the four libraries. The vast majority of mapped reads uniquely matched to unigenes (> 68%), and the percentage of multi-position matched reads was less than 15% (Table 4).

**Table 4:** Summary of reads mapped to reference genes of the *de novo* assembly of transcriptome. NH, Normal nitrogen level and high temperature treatment; NN, Normal nitrogen level and normal temperature treatment; HH, High nitrogen level and high temperature treatment; HN, High nitrogen level and normal temperature treatment. Sample IDs were labeled as follows: “G” for the strain of rice used (Ganxin 203), four treatment combinations and a number for the two pooling duplicates.

Samples	Total reads	Total base pairs	mapped reads	Perfect match	< = 2 bp Mismatch	Unique match	Multi-position match	Total unmapped reads
GHH-1	11,982,487 (100.00%)	587,141,863 (100.00%)	9,966,553 (83.18%)	8,211,092 (68.53%)	1,755,461 (14.65%)	8,285,750 (69.15%)	1,680,803 (14.03%)	2,015,934 (16.82%)
GHH-2	11,779,163 (100.00%)	577,178,987 (100.00%)	9,926,836 (84.27%)	8,415,848 (71.45%)	1,510,988 (12.83%)	8,199,782 (69.61%)	1,727,054 (14.66%)	1,852,327 (15.73%)
GHN-1	11,678,856 (100.00%)	572,263,944 (100.00%)	9,714,147 (83.18%)	8,163,006 (69.90%)	1,551,141 (13.28%)	8,133,912 (69.65%)	1,580,235 (13.53%)	1,964,709 (16.82%)
GHN-2	11,971,075 (100.00%)	586,582,675 (100.00%)	9,947,698 (83.10%)	8,322,389 (69.52%)	1,625,309 (13.58%)	8,238,668 (68.82%)	1,709,030 (14.28%)	2,023,377 (16.90%)
GNH-1	11,791,352 (100.00%)	577,776,248 (100.00%)	9,974,328 (84.59%)	8,216,291 (69.68%)	1,758,037 (14.91%)	8,283,020 (70.25%)	1,691,308 (14.34%)	1,817,024 (15.41%)
GNH-2	12,062,967 (100.00%)	591,085,383 (100.00%)	10,089,996 (83.64%)	8,382,302 (69.49%)	1,707,694 (14.16%)	8,429,651 (69.88%)	1,660,345 (13.76%)	1,972,971 (16.36%)
GNN-1	11,656,703 (100.00%)	571,178,447 (100.00%)	9,600,579 (82.36%)	7,905,941 (67.82%)	1,694,638 (14.54%)	7,980,916 (68.47%)	1,619,663 (13.89%)	2,056,124 (17.64%)
GNN-2	11,739,441 (100.00%)	575,232,609 (100.00%)	9,766,302 (83.19%)	8,088,274 (68.90%)	1,678,028 (14.29%)	8,232,714 (70.13%)	1,533,588 (13.06%)	1,973,139 (16.81%)

### Assessment of DGE sequencing

In order to evaluate the DGE data, we performed a saturation analysis to detect positive associations with the number of detected genes and the total read number. As showed in S7 Fig, the DGE libraries were saturated at 10M sequences for each of the four DGE libraries.

We used the distribution of reads on the reference genes to evaluate the randomness. Since reference genes are in different lengths, the reads position on each gene was standardized to a relative position, which was calculated as the ratio between reads location position on the gene and gene length, then the number of reads in each position was counted. The randomness analysis indicated that the results were of good quality, and that the reads in every position were evenly distributed (S8 Fig).

The next evaluated gene coverage, as determined by the percentage reads that mapped to a gene. The value was determined as the ratio of the base number in a gene covered by mapping unique reads to the total number of bases in the gene. About 30% of the samples showed gene coverage above 90% % (S9 Fig). We used the reads per kb per million reads method (RPKM) method to determine gene expression levels. The summary results of gene expression and related information for all examples are provided in S4 Table.

Comparing DEGs require a high correlation among the same replicates. We used Pearson’s method to obtain the coefficient of all genes between every two replicates. The coefficient of all genes between every two samples was above 80%, which suggested that the results between the two replicates were consistent (S10 Fig).

### Screening of differentially expressed genes (DEGs)

**Fig 4:**
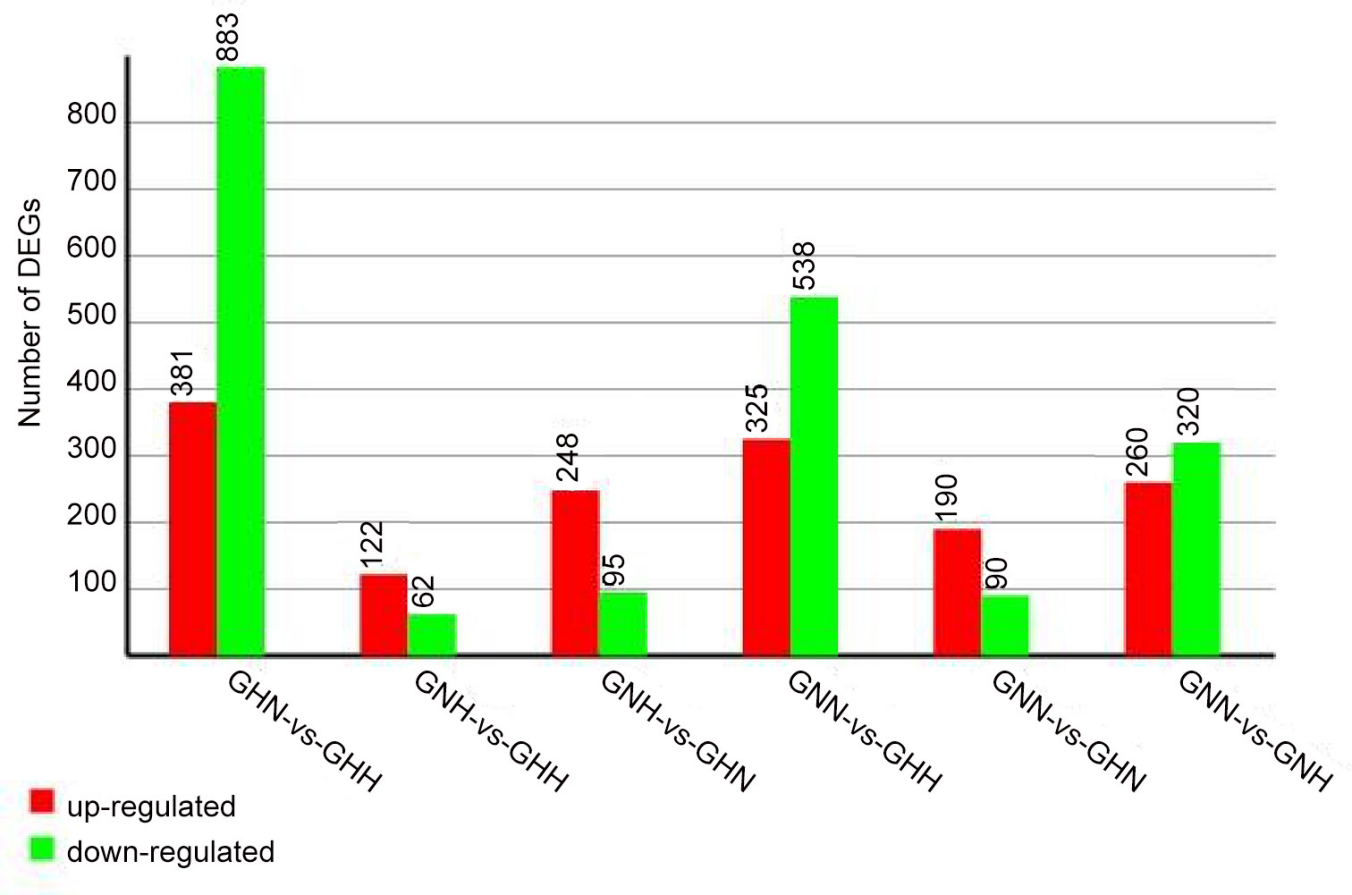
Chart listing statistics of the groups of differentially expressed genes. The differentially expressed genes were identified with the criteria (twofold or more change and P< 0.001).

We analyzed DEGs between different treatments to identify genes with a significant change in expression in response to heat or nitrogen stress. We used a twofold or more change and P< 0.001 as our significance cut-off. Comparing differences in temperature treatment in high-nitrogen treatment background (HN vs HH libraries), we identified 1,264 differentially expressed unigenes, with 381 upregulated and 883 downregulated unigenes. We identified 122 upregulated and 62 downregulated DEGs (total 184) when comparing different nitrogen treatments in high-heat background (NH vs HH). Different nitrogen treatments in normal temperature background (NN vs HN) showed a total of 280 DEGs, with 190 upregulated and 90 downregulated unigenes. Lastly, comparing different heat treatments in a normal nitrogen level background (NN vs NH) resulted in a total of 580 DEGs, including 260 upregulated and 320 downregulated unigenes (Fig 4, S5 Table). Interestingly, many of the genes identified in this comparison were unannotated, or annotated as “hypothetical protein,”, therefore their functions are unknown (S5 Table).

Of the DEGs responding to temperature that were recovered in different heat treatments, 70 DEGs from NN vs NH and 135 DEGs from HN vs HH had annotated functions (S6 Table). These genes were principally associated with calcium-dependent protein kinase, cytochrome P450, flavonoid, heat shock protein, peroxidase, ubiquitin, photosynthesis, chlorophyll biosynthetic process, zinc transporter, transcription factor, sporopollenin biosynthetic process, and pollen exine formation etc. (S6 Table). Meanwhile, 17 and seven DEGs responding to nitrogen were focused between GNN and GHN, and between GNH and GHH, respectively (S6 Table). These genes were principally related to glutamine synthetase, ubiquitin, leucine-rich repeat family protein, amino acid transporter, pollen development, and hormone etc. (S6 Table).

**Fig 5:**
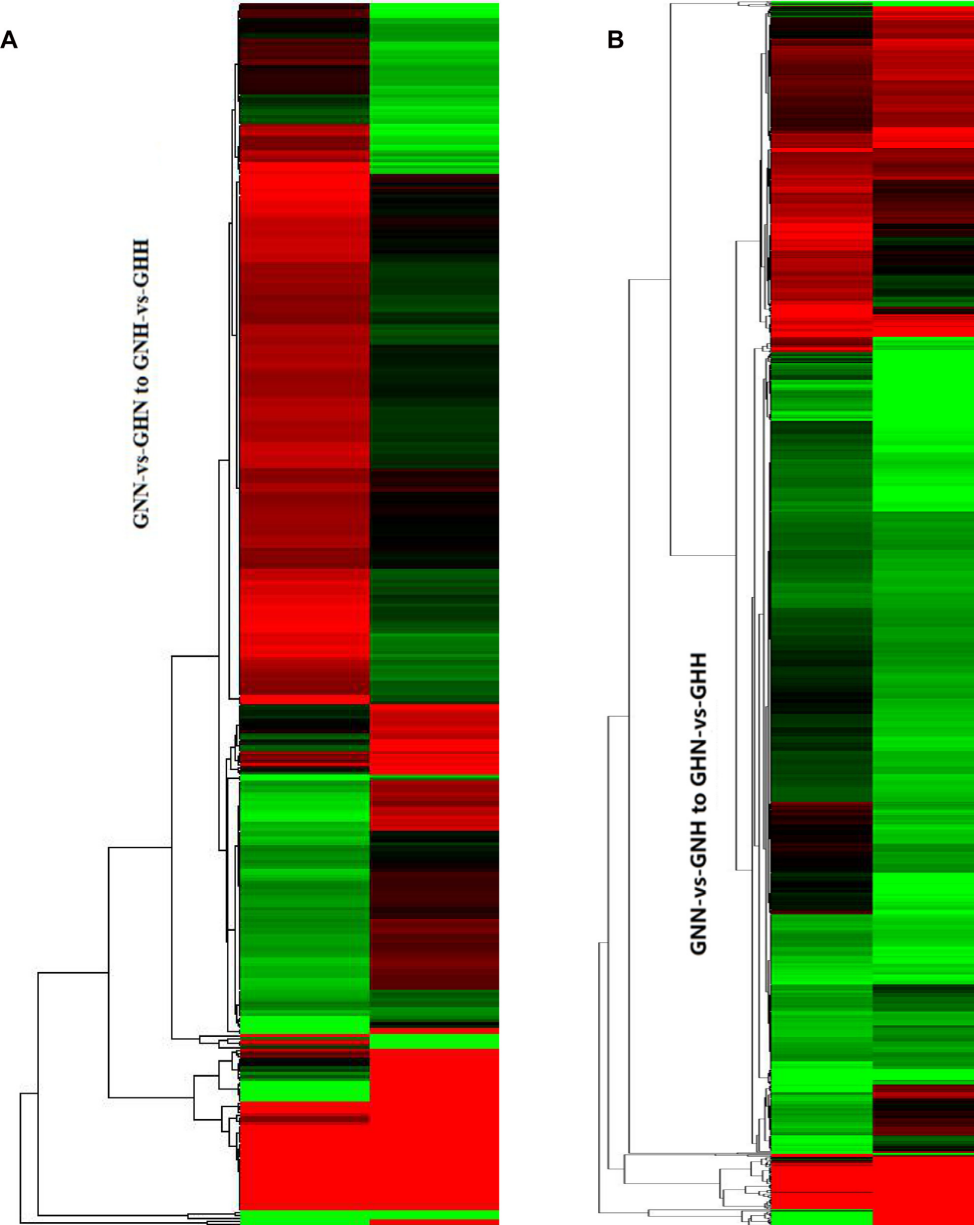
Cluster image of differentially expressed genes levels. Each column represents an experimental condition, and each row represents a gene that is differentially expressed. The log2 (RPKM) of differentially expressed genes were clustered, and red indicates upregulation and green indicates downregulation. Genes labeled with color closer to red than green, indicates a more highly expressed gene.

In order to further understand the combined effects of temperature and nitrogen, we compared the DEGs in different treatment combinations. A total of 189 DEGs were shared between two comparisons (GNN-vs-GNH and GHN-vs-GHH), and 12 DEGs were co-expressed in the GNN-vs-GNH and GHN-vs-GHH comparisons (S7 Table). A total of 27 and 12 DEGs associated with temperature and nitrogen were identified (Table 5), and the hierarchical clustering of the DEGs based on the log_2_ (RPKM) values under the same nitrogen level and the same temperature was showed in Fig 5A and 5B.

**Table 5:** Summary of differentially expressed genes that occurred simultaneously in different samples. All genes in this table were annotated by KEGG, GO, and BLAST NR databases, and showed differential expression with probability greater than or equal to 0.8 and |log_2_Ratio| greater than or equal to 1. Unigenes with positive log_2_Ratio represent upregulated, and genes with negative log_2_Ratio represent downregulated genes.

Gene ID	log_2_Ratio(GNH/GNN)	log_2_Ratio(GHH/GHN)	Gene annotation by KEGG orthology, GO process, or BLAST NR
**GNN-vs-GNH and GHN-vs-GHH**			
Unigene31305	-1.8074	-2.0621	cytochrome P450, family 86, subfamily A
CL5877.Contig2	-2.5896	-2.3829	flavonoid biosynthetic process
CL2576.Contig1	2.6524	3.3838	HSP20 family protein
Unigene27491	3.2021	3.4331	HSP20 family protein
Unigene47356	-2.5550	1.6339	response to heat
CL628.Contig1	4.3822	5.1891	response to heat
CL3536.Contig1	-1.9360	-1.7974	peroxidase
Unigene28945	-1.7963	-1.9143	sporopollenin biosynthetic process
Unigene1573	-1.6245	-2.0675	sporopollenin biosynthetic process
Unigene44325	-3.2040	-2.2430	pollen exine formation
Unigene44338	-2.4144	-2.0983	pollen exine formation
Unigene44326	-2.2904	-1.8276	pollen exine formation
Unigene44341	-1.8817	-1.6191	pollen exine formation
Unigene44342	-1.7109	-1.6853	pollen exine formation
CL2537.Contig2	-2.4259	-2.2329	pollen maturation; regulation of photosynthesis
CL1622.Contig2	-2.6821	-3.3782	pollen tube growth; pollen development
CL5831.Contig3	-1.9417	-2.0897	regulation of flower development
Unigene33050	-1.8636	-2.9267	response to auxin stimulus
Unigene16848	-1.6707	-2.8885	response to gibberellin stimulus
Unigene28134	-2.4774	-1.9253	cytokinin receptor
Unigene1331	-2.2649	-2.1603	meiotic serine proteinase-like protein
CL1141.Contig2	1.7047	1.6305	ubiquitin C
Unigene24097	-2.8501	-4.2379	ubiquitin-dependent protein catabolic process
Unigene30754	-2.6481	-3.0905	ubiquitin-dependent protein catabolic process
CL3844.Contig2	-2.0309	-3.2847	ubiquitin-dependent protein catabolic process
Unigene15197	-1.9490	-2.2725	upstream-binding transcription factor
Unigene15196	-1.9023	-1.8655	upstream-binding transcription factor
Gene ID	log_2_Ratio(GHN/GNN)	log_2_Ratio(GHH/GNH)	Gene annotation by KEGG orthology, GO process, or BLAST NR
**GNN-vs-GHN and GNH-vs-GHH**			
CL3845.Contig2	-2.0665	2.4105	unknown
CL3845.Contig1	-2.0470	1.7900	unknown
CL7441.Contig2	-1.8205	2.0338	unknown
CL5161.Contig1	-2.5637	2.2662	unknown
Unigene40707	-2.8156	2.0969	unknown
CL5161.Contig2	-2.3986	2.0103	unknown
Unigene45035	-3.7738	3.3847	unknown
Unigene14123	-12.8103	11.0292	unknown
Unigene57543	-8.7199	6.8795	unknown
Unigene55829	2.2879	-2.8875	Os12g0428700
Unigene9754	2.6812	-2.3095	hypothetical protein OsI_38816
Unigene53453	-2.4920	1.8284	cytochrome b6-f complex subunit 8

To understand the functions of these differentially expressed genes, all the DEGs were mapped to the GO database. The DEGs were categorized into smaller functional groups in the three main GO categories (biological process, cellular component, and molecular function) (S8 Table, S11 Fig). Based on sequence homology, the unigenes annotated in the GO database were categorized into the 49 functional groups in GNN-vs-GNH and GHN-vs-GHH, and 40 and 35 functional groups in GNN-vs-GHN and GNH-vs-GHH, respectively. Among these groups, “metabolic process” and “cellular process” were the dominant subcategories within the “biological process” category, the “cell” and “cell part” subcategories were dominant in the “cellular component” category, and “catalytic activity” and “binding” were the dominant subcategories in the “molecular function” category (S11 Fig).

**Fig 6:**
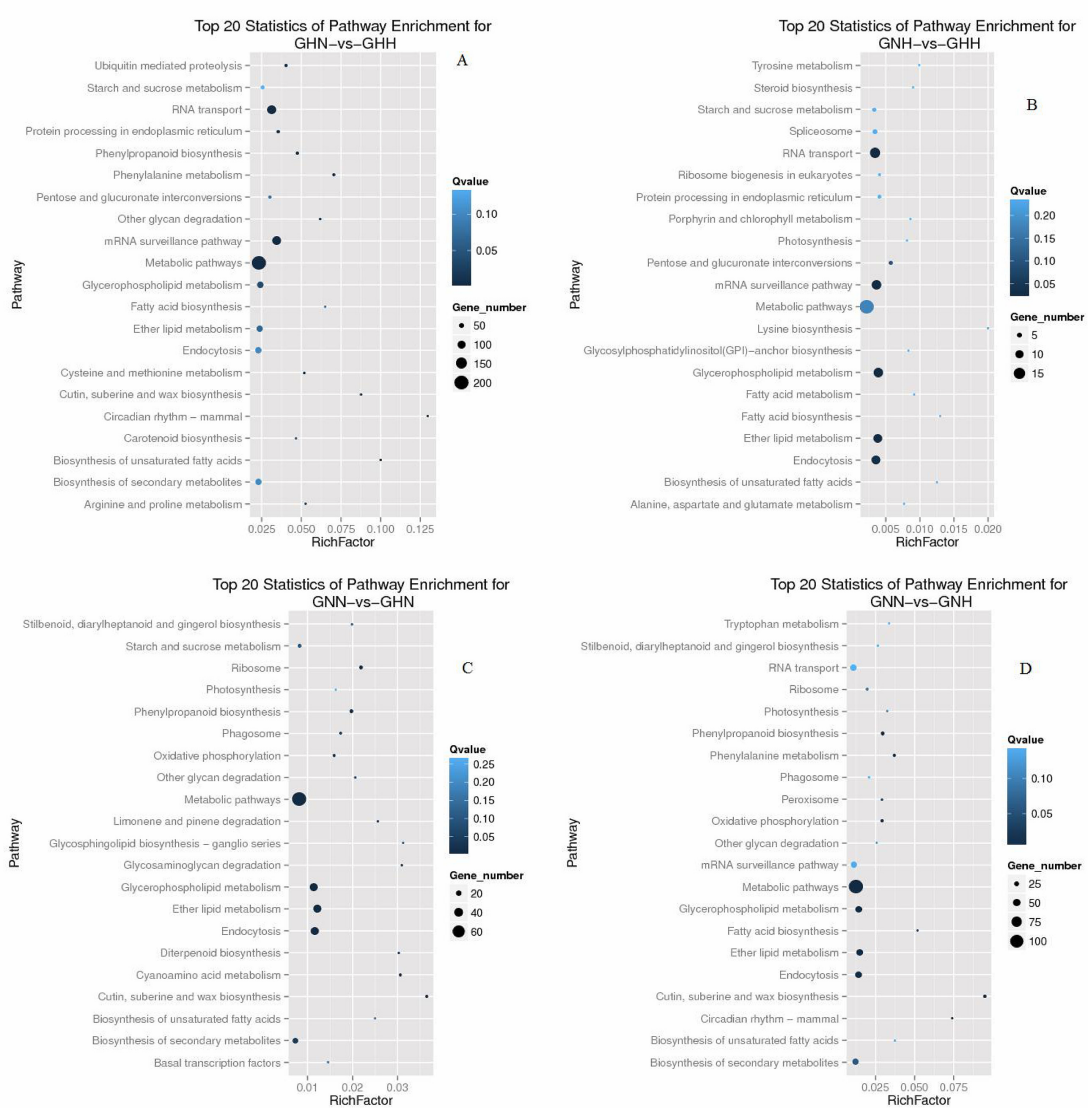
KEGG pathway classification of differentially expressed genes. RichFactor is the ratio of DEG numbers annotated in a given pathway term to all gene numbers that were annotated in the pathway term. Greater RichFactor means greater intensiveness. Q-value is the corrected P-value ranging from 0~1, and lower Q-value indicates greater intensiveness. The top 20 pathway terms enriched by the KEGG database are listed in this figure.

To further understand the functions of DEGs, we mapped all the DEGs to terms in the KEGG database and found many pathways to be significantly enriched. For GNN-vs-GNH and GHN-vs-GHH, 256 and 47 unigenes had KO IDs and could be categorized into 82 and 29 pathways, respectively. Of those, 5 and 11 pathways were significantly enriched (Q value< 0.05), and genes involved in metabolic pathways were the most significantly enriched (S9 Table). For GNN-vs-GHN and GNH-vs-GHH, 130 and 564 unigenes had KO IDs that were categorized into 59 and 86 pathways, respectively. Of those, 14 and 15 pathways were significantly enriched (Q value< 0.05), and genes involved in metabolic pathways were the most significantly enriched (S9 Table). The top 20 KEGG pathways of DEGs in these four comparisons mentioned above are listed in Fig 6A–6D.

### Validation of gene expression by RNA-seq

**Fig 7:**
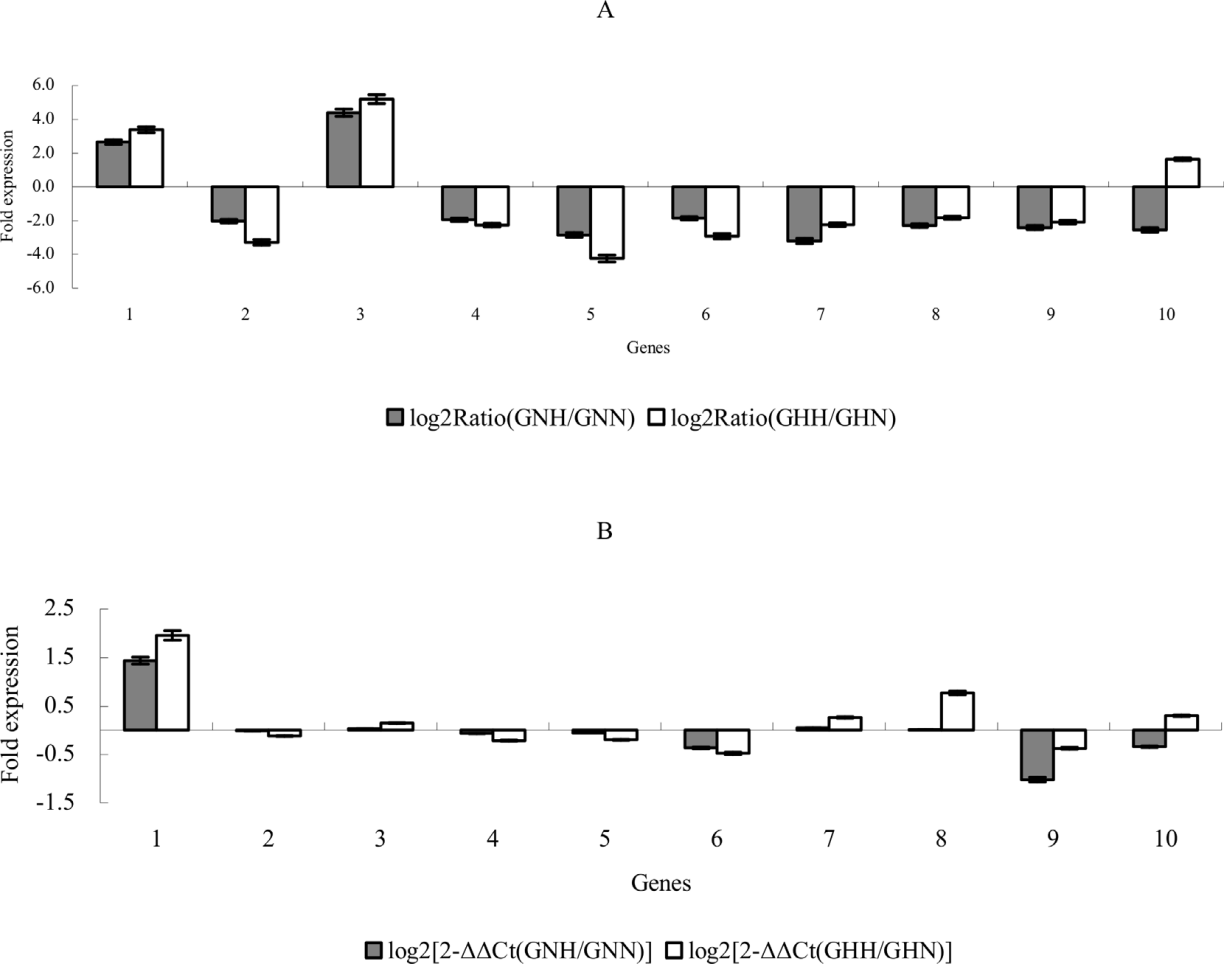
Expression pattern of the selected genes by RT-qPCR and RNA-seq analysis. (A) Gene expression data for RNA-seq analysis. (B) The RT-qPCR analysis of gene expression data. The fold changes of the genes are shown on the y-axis.

To validate the expression profiles obtained by RNA-seq, we performed RT-qPCR analysis of 10 randomly selected DEGs (S1 Table). For all 10 genes, we found the same expression profiles as the original RNA-seq data (Fig 7), suggesting that the RNA-seq data obtained for the DEGs analysis was credible.

### Effects of nitrogen level and high temperature at late spikelet differentiation stage (meiosis) on spikelets fertility of rice

**Fig 8:**
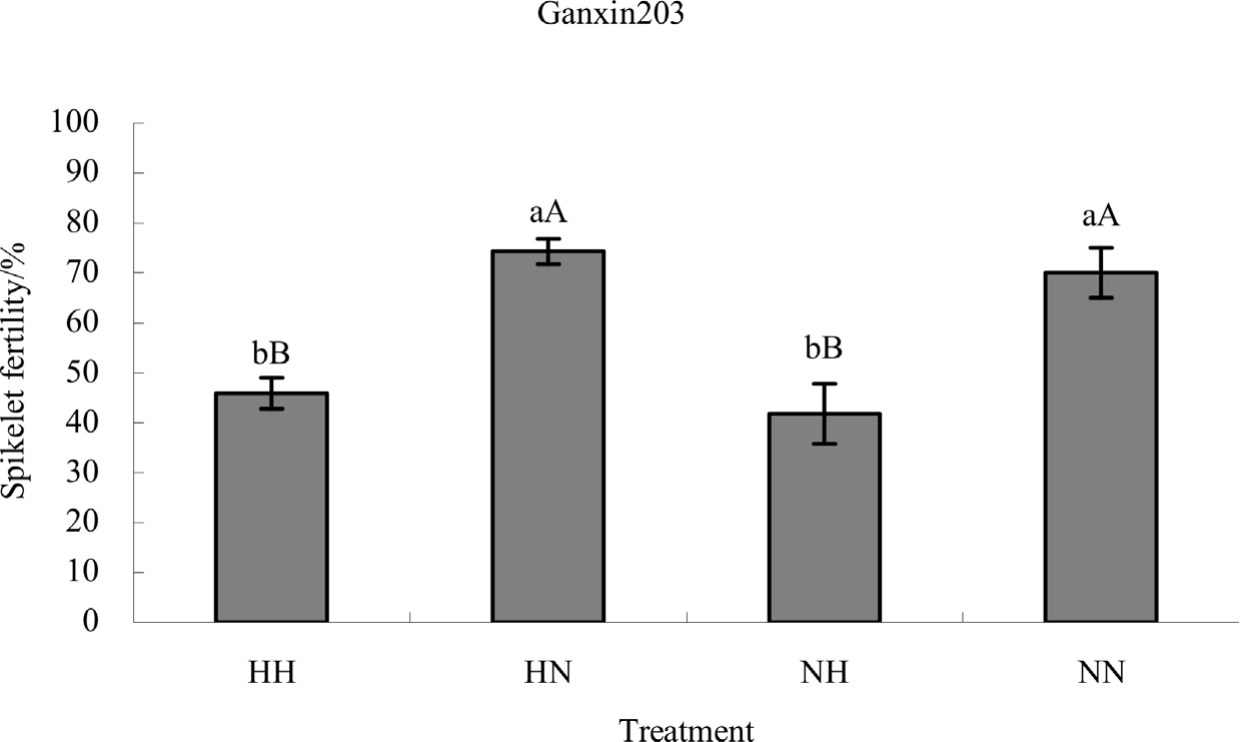
Effects of nitrogen level and high temperature at late spikelet differentiation stage on spikelet fertility of rice. Bargraph data show as mean ± standard error. Bars marked with the same letters indicate no significant difference at 5% level or 1% level. NH, Normal nitrogen level with high temperature; NN, Normal nitrogen level with normal temperature; HH, High nitrogen level with high temperature; HN, High nitrogen level with normal temperature.

To test the effects of nitrogen level and high temperature on spikelet fertility, we investigated the spikelet fertility in the four different treatment combinations. High temperature at late spikelet differentiation stage significantly decreased spikelet fertility of Ganxin203 compared with normal temperature at both high nitrogen levels and normal nitrogen levels (p< 0.01). The reduction in spikelet fertility in response to high temperature was less in high nitrogen levels compared to normal nitrogen levels (Fig 8). These results suggest that appropriate higher nitrogen levels can mitigate the negative the effects of high temperature on spikelet fertility.

## Discussion

RNA-seq is a powerful and efficient tool for mining genes that are related to specific functions. In this study, we performed *de novo* assembly of transcriptome in rice spikelet, and obtained a total 4.70 Gb of transcriptome data. We obtained over 10 million clean reads from the samples of four digital gene expression profiling libraries (NH, NN, HH, HN), and found many upregulated and downregulated DEGs among the libraries. The DEGs data provided comprehensive gene expression information for the rice spikelet, facilitating our understanding of the molecular mechanisms of rice spikelet in response to temperature interacting with nitrogen during meiosis.

High temperature is a major abiotic stress limiting rice growth and development, and rice alters gene expression in response to heat stress to increase the heat tolerance. The heat shock proteins (HSPs) including HSP100, HSP90, HSP70, HSP60, HSP40 and HSP20, and small heat shock proteins (sHSPs) play a key role in protecting plant cells and tissues from thermal or oxidative stress [48,49,50,51]. The loss-of-function mutant of rice cytosolic HSP70 gene (*OsctHSP70-1*) did not show a clear defective phenotype under high temperature, and this is likely because of the existence of another gene family member that is closely clustered with *OsctHSP70-1*, which showed compensatory increase in expression [52]. The HSP70s accumulate in response to a rapid rise in temperature [53,54]. *AtHSP70-15*-deficient plants under heat stress result in drastic increase in mortality, indicating that *AtHSP70-15* plays an essential role during normal growth and in the heat response of *Arabidopsis* [55]. The heat-tolerant rice variant N22 shows upregulation of a cold (putative) and a heat (unknown) shock protein under 38°C during anthesis, indicating that heat shock proteins are likely to contribute to the heat tolerance of the N22 variant [7]. We found three DEGs annotated as HSPs with two upregulated HSP20s and one downregulated HSP70 when comparing heat treatments in normal nitrogen levels (NN vs NH), and nine upregulated DEGs annotated as HSPs with four HSP20s, four HSP70s and one HSP90 when comparing heat treatments in high nitrogen levels (HN vs HH) (S6 Table). Interestingly, there were two HSP20s (*CL2576*.*Contig1*, *Unigene27491*) and two heat-responsive genes (*Unigene47356*, *CL628*.*Contig1*) that were recovered in both comparisons (Table 5). The gene expression (log_2_Ratio value) of these four co-expressed DEGs were higher in the high nitrogen experiments (HN vs HH) compared to normal nitrogen (NN vs NH), indicating that plants grown in higher nitrogen levels may show better heat tolerance through elevating HPSs gene expression.

The cytochrome P450 family and ubiquitin protein genes play an important role during normal growth, development, and also show a response to heat in rice plants. A previous study found that the cytochrome P450 CYP4/CYP19/CYP26 subfamilies gene (*Os12g05440*), ubiquitin-protein ligase UBC gene (*Os06g30970*), and E3 ubiquitin ligase DNA binding like gene (*Os03g45730*) showed significant changes (>16 fold) after 10 minutes of 42°C heat stress [56]. Most E3 ligase genes show upregulation [10], while most heat-responsive (P450 family) genes are repressed in response to heat stress, [10]. SIZ1, a SUMO E3 ligase, responds to both high and low temperature [57], and controls spikelet fertility through regulation of anther dehiscence [58]. High temperature (39°C) at the microspore stage reduces spikelet fertility of rice, and represses cytochrome P450 family genes in the anther [6]. In the present study, we observed that two E3 ubiquitin-protein ligase genes and four ubiquitin-conjugating enzyme genes were upregulated, five cytochrome P450 family genes and six genes involved in ubiquitin-dependent protein catabolic process were downregulated when comparing different temperature treatments in the plants grown in normal nitrogen levels (NN vs NH) (S6 Table). We identified three cytochrome P450 family genes that were downregulated, four E3 ligase genes, of which one was upregulated and three were downregulated, eight ubiquitin-conjugating enzyme genes with one upregulated and seven downregulated, and 11 genes annotated as ubiquitin-dependent protein catabolic process with four upregulated and seven downregulated in the different temperature treatments at high nitrogen levels (HN vs HH) (S6 Table). Interestingly, there were three genes annotated as ubiquitin-dependent protein catabolic process (*Unigene24097*, *Unigene30754*, and *CL3844*.*Contig2*), one ubiquitin-conjugating enzyme gene (*CL1141*.*Contig2*), one P450 family gene (*Unigene31305*) that were identified in both comparisons, but the expression of these genes were higher in the high nitrogen treatment (Table 5). Given that these genes were recovered in both conditions, they may represent genes that are vital for heat response in the rice spikelet.

Spikelet fertility is closely related to pollen exine formation, pollen tube growth, pollen maturation, and pollen development in plants. The ABC transporter gene *OsABCG15* regulates pollen development in rice, and Wu et al [59] showed that *OsABCG15* plays an essential role in the formation of the rice anther cuticle and pollen exine. Ling et al [60] identified 291 mature anther-preferentially expressed genes (*OsSTA*) in rice, and these OsSTA genes were associated with pollen fertility, pollen germination and anther dehiscence in rice. The *pollen semi-sterility1* (*PSS1*) encodes a kinesin-1-like protein to regulate anther dehiscence in rice [61]. The RING-type E3 ubiquitin ligase, POLLEN TUBE BLOCKED 1 (PTB1), positively regulates rice panicle seed setting rate by promoting pollen tube growth [62]. OsAP65, a rice aspartic protease, is essential for male fertility and plays a role in pollen germination and pollen tube growth [63]. Chueasiri et al [64] quantified gene expression in anthers of temperature-sensitive rice plants grown in controlled growth rooms (26°C and 32°C) in fertile and sterile conditions, showing that plant orosomucoids-like proteins influence sphingolipid homeostasis, and deletion of this gene affects spikelet fertility resulting from abnormal pollen development. The transcription factor bHLH142 plays as a pivotal role in tapetal programmed cell death and pollen development during early meiosis in rice [65]. In this study, we found nine spikelet genes annotated using the GO database including five pollen exine formation, one pollen maturation, and three sporopollenin biosynthetic process, all of which were downregulated in the different heat treatments in normal nitrogen treatment (NN vs NH) (S6 Table). For the different heat treatments in high nitrogen conditions (HN vs HH), we found the downregulation of spikelet genes including one anther development, one pollen tube growth, one pollen maturation, six pollen exine formation, and seven sporopollenin biosynthetic process (pollen exine formation) (S6 Table). Moreover, we found that five pollen exine formation, one pollen tube growth, one pollen maturation, and two sporopollenin biosynthetic process genes were co-expressed (Table 5). Interestingly, we found higher log_2_Ratio value in the plants grown in high nitrogen in one pollen maturation gene (*CL2537*.*Contig2*), and five pollen exine formation genes (*Unigene44342*, *Unigene44326*, *Unigene44325*, *Unigene44341*, and *Unigene44338*) (Table 5). The results suggest that these co-expressed genes play important roles in rice spikelet development in response to high temperature at high nitrogen levels. Accordingly, spikelet fertility of rice was significantly reduced under high temperature, but less reduced under high nitrogen treatment (Fig 7). Taken together, our results indicate that significant expression changes of genes involved in pollen maturation and pollen exine formation may have important roles in maintaining the cellular homeostasis to cope with heat stress at high nitrogen level in rice spikelet. In addition, we found 70 DEGs with 22 upregulated and 48 downregulated genes, and 135 DEGs with 35 upregulated and 100 downregulated genes in response to high temperature in NN vs NH and HN vs GHH, respectively (S6 Table), indicating that gene expression changes in response to high temperature is more robust at high nitrogen level.

Plant growth and development is often subjected to nitrogen limitation. To cope with this, plants have evolved adaptive strategies to successfully complete their life cycles [13,66]. Peng et al [16] found that nitrogen limitation alters the expression levels of 629 genes with 340 upregulated and 289 downregulated in *Arabidopsis*, and the downregulated genes include those involved in photosynthesis and the synthesis of nitrogenous macromolecules such as chlorophyll, proteins, amino acids and nucleotides. Lian et al [17] subjected rice to low nitrogen stress, then analyzed the expression profiles using a microarray of 11,494 rice ESTs representing 10,422 unique genes. These differentially expressed genes identified in this study were mainly involved in photosynthesis, energy metabolism, transcription factors regulatory, and signal transduction. Phytohormones play many biological roles in higher plants, and genes required for IAA and gibberellin (GA) synthesis are coordinately expressed during later microspore/pollen (MS/POL) development [67]. In contrast, genes for GA signaling are expressed early MS/POL development. Application of auxin can block the transcriptional activity, which leads to the production of normal pollen grains, and the normal seed setting rate even under increasing temperatures [68]. Plants that are GA-deficient show defective in pollen tube elongation, resulting in a low spikelet fertilization frequency, whereas GA-insensitive semidominant mutants are mainly defective in viable pollen production. GA biosynthesis genes are preferentially expressed after meiosis during pollen development [69], and there is a reduction of gibberellin in plants subjected to low temperature, which disrupts pollen development and causes severe reduction of seed setting in rice [70]. In our results, we identified 17 and seven nitrogen-responsive DEGs in normal temperature conditions (NN vs HN) and in high temperature conditions (NH vs HH), respectively (S6 Table). The nitrogen-responsive genes were mainly involved in pollen development and plant hormone (auxin, gibberellin, abscisic acid, and ethylene). Furthermore, a total of 12 DEGs, of which nine genes had no functional annotation, were co-expressed, and we did not find any gene that was responsive to nitrogen in the two comparisons (Table 5). Therefore, the nitrogen levels may not have a dominant effect in our study.

## Supporting Information

S1 Fig The Pipeline of experiments prior to Illumine sequencing. (TIF)

S2 Fig The Pipeline of bioinformatics analysis of the transcriptome. (TIF)

S3 Fig The process of unigenes assembly. (TIF)

S4 Fig Scheme of standard bioinformatics analysis of RNA-Seq (Quantification). (TIF)

S5 Fig The distribution of NR annotation results. Figures show (A) E-value distributions, (B) similarity (identity) distributions, and (C species distributions. (TIF)
(TIF)

S6 Fig The quality assessment of reads. The numbers and percentage of reads containing adaptor, containing N, low quality reads, and clean reads, are shown. (TIF)
(TIF)

S7 Fig The sequencing saturation analysis. (TIF)

S8 Fig The randomness assessment. (TIF)

S9 Fig The gene coverage. (TIF)

S10 Fig The correlation analysis of Pearson coefficient in gene expression between the two pooled sample duplicates. (TIF)

S11 Fig The GO classification analysis of differentially expressed genes (DEGs). (TIF)

S1 Table List of primers used in real-time quantitative PCR analysis. (XLS)

S2 Table The quality control of RNA used in digital gene expression (DGE) sequencing. (XLS)

S3 Table The summary of mapping results showing reads mapped to reference genome (*Indica* rice). (XLS)

S4 Table The quantification of gene expression. (RAR)

S5 Table The differentially expressed genes (DEGs) identified in different samples. (XLS)

S6 Table The differentially expressed genes (DEGs) in response to temperature, and nitrogen in different samples. (XLS)

S7 Table The summary of differentially expressed genes (DEGs) that were found co-expressed in different samples. (XLS)

S8 Table The GO enrichment analysis of the differentially expressed genes (DEGs). (XLS)

S9 Table The KEGG pathway enrichment analysis of the differentially expressed genes (DEGs). (XLS)
