# Supplementary figures and images for: Using RNA-seq to Profile Gene Expression of Spikelet Development in Response to Temperature and Nitrogen during Meiosis in Rice (Oryza sativa L.)
Source: PLoS One. 2015 Dec 29;10(12):e0145532. doi: 10.1371/journal.pone.0145532 (PMC4694716; doi:10.1371/journal.pone.0145532)

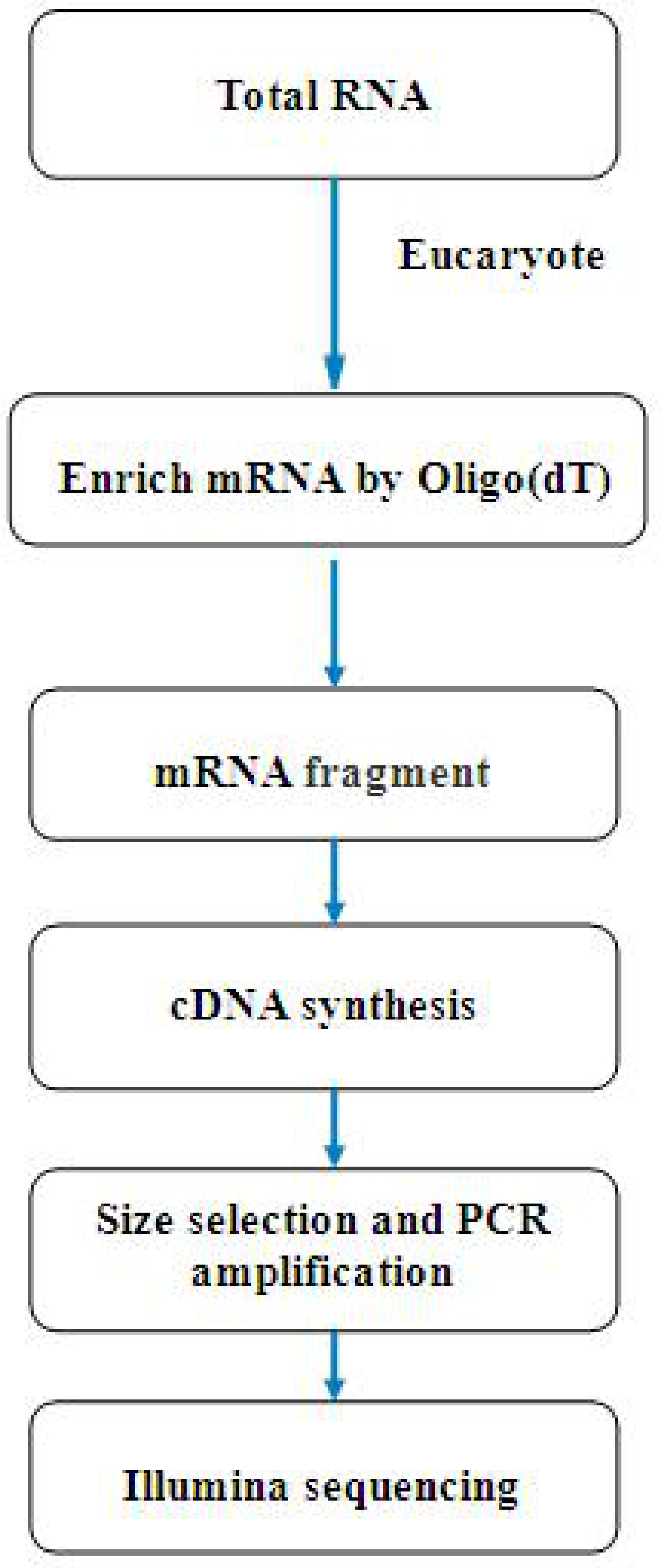

Supplement: S1 Fig — (TIF) [file pone.0145532.s001.tif]

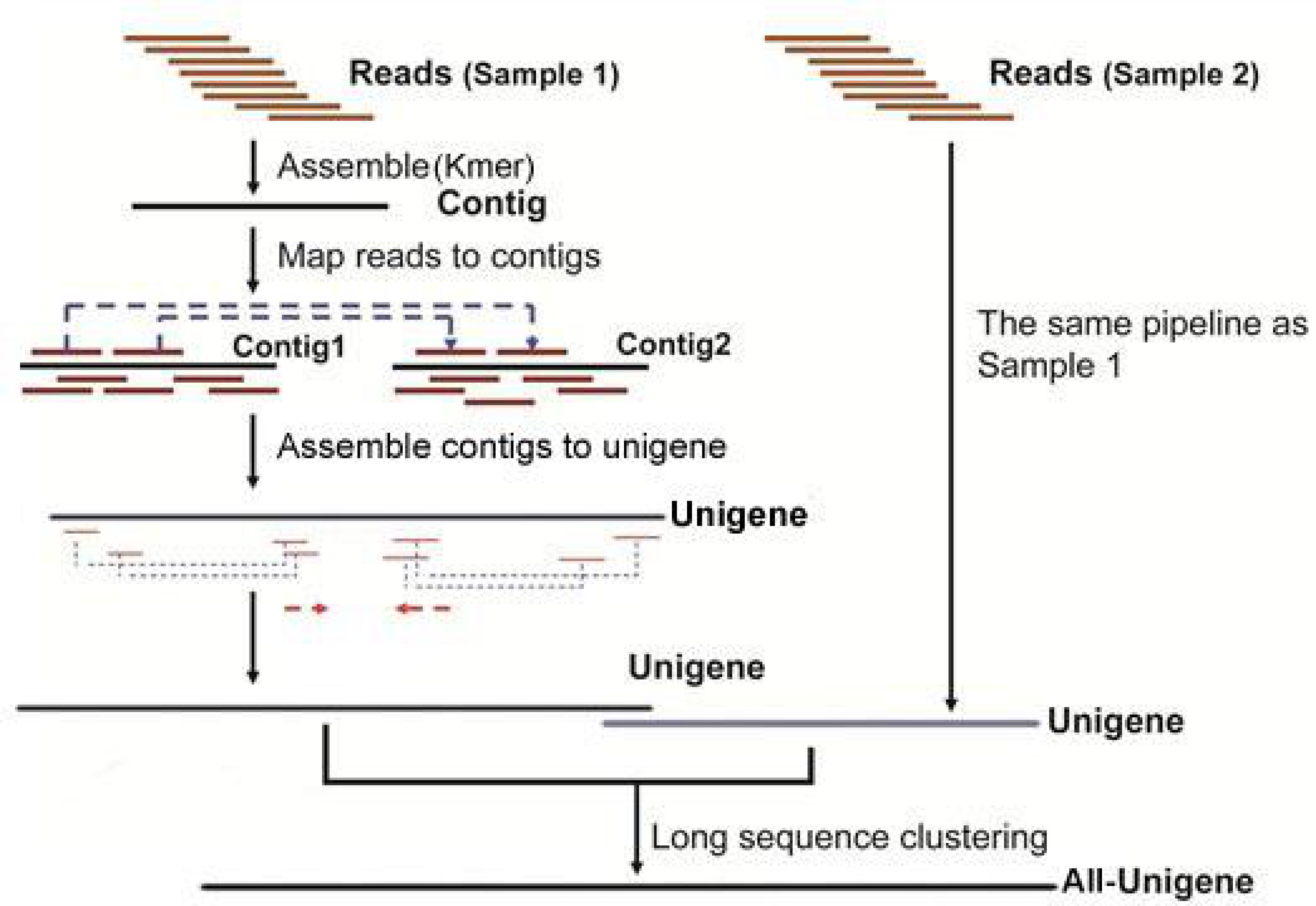

Supplement: S2 Fig — (TIF) [file pone.0145532.s002.tif]

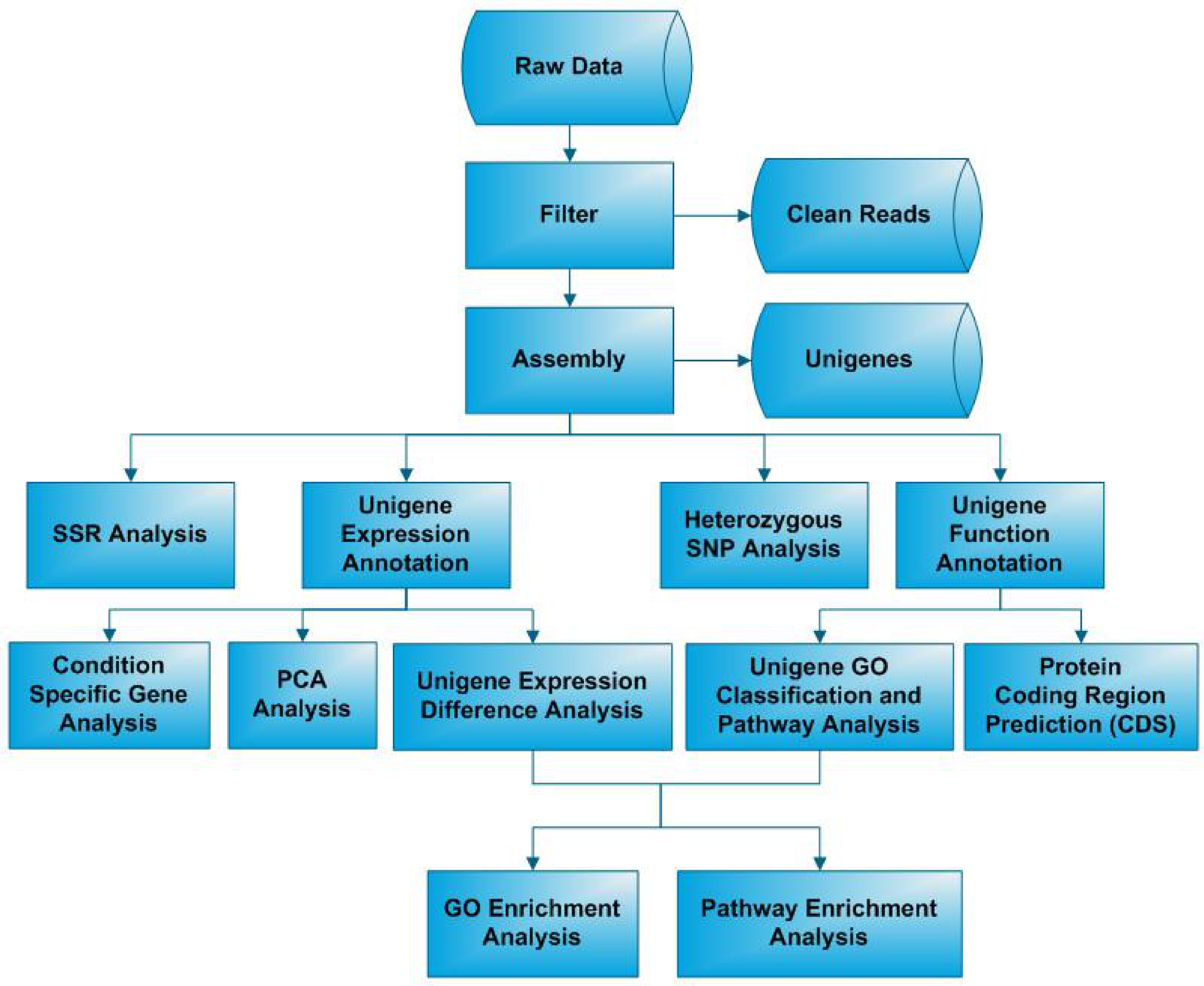

Supplement: S3 Fig — (TIF) [file pone.0145532.s003.tif]

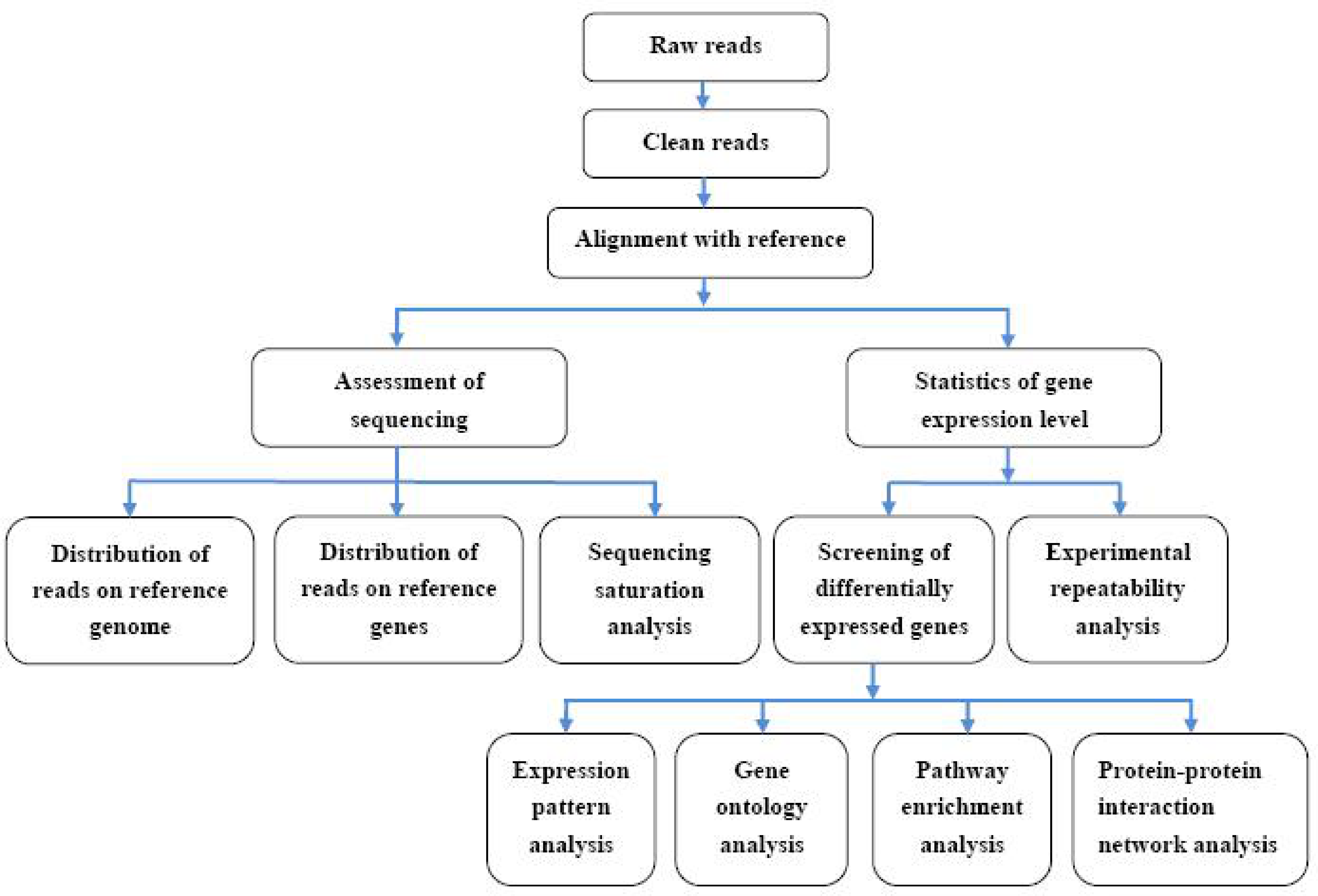

Supplement: S4 Fig — (TIF) [file pone.0145532.s004.tif]

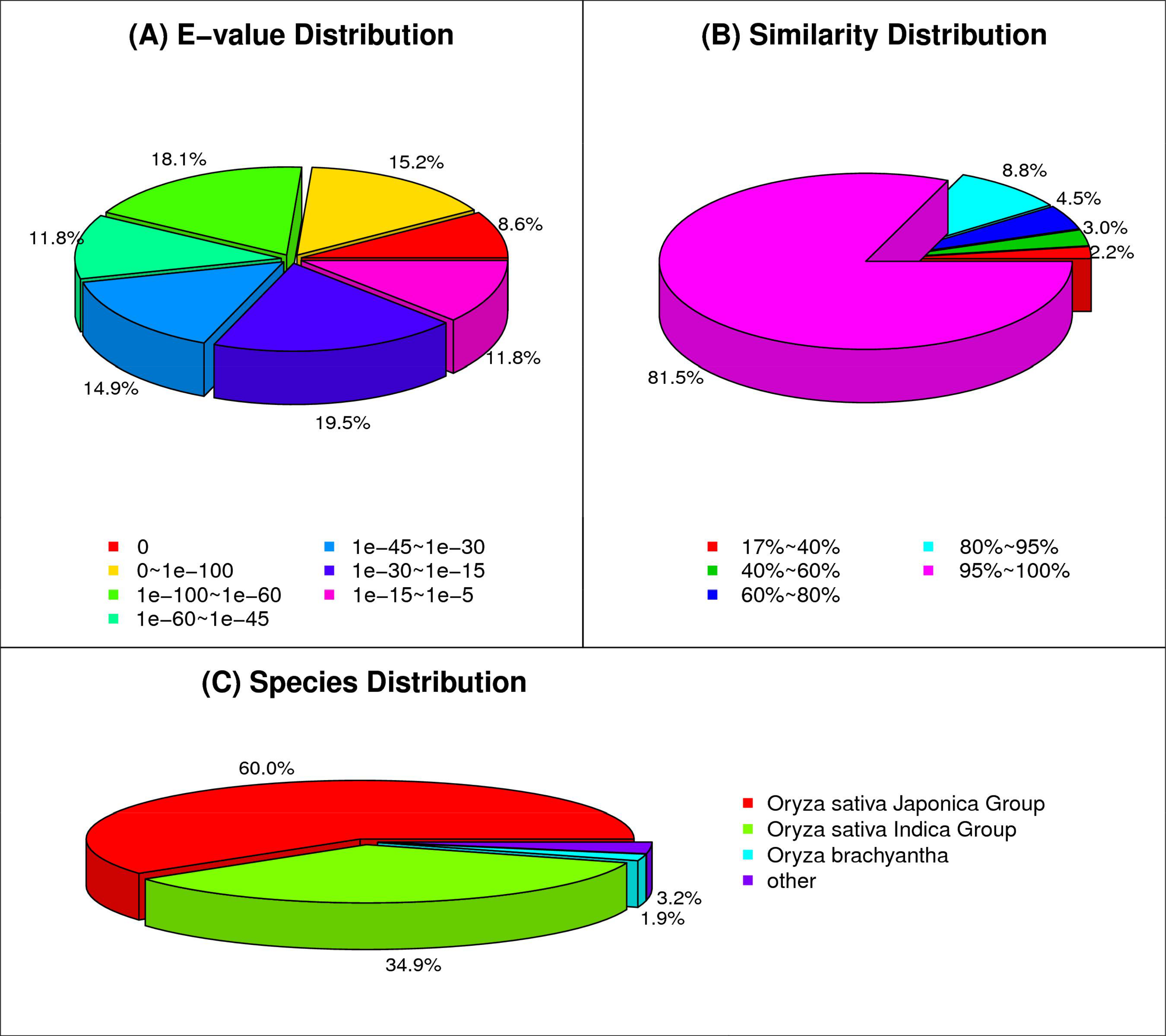

Supplement: S5 Fig — Figures show (A) E-value distributions, (B) similarity (identity) distributions, and (C species distributions. (TIF) (TIF) [file pone.0145532.s005.tif]

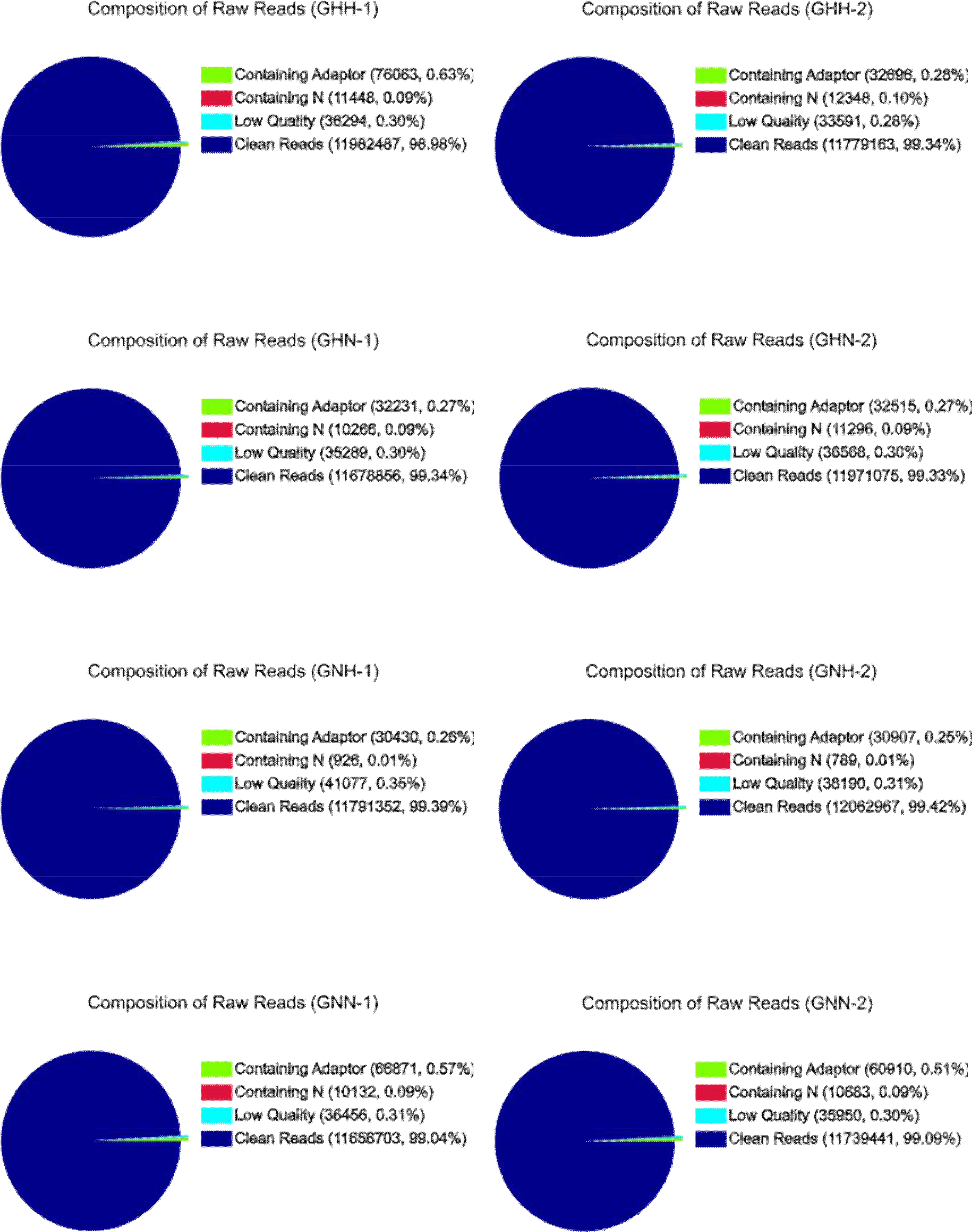

Supplement: S6 Fig — The numbers and percentage of reads containing adaptor, containing N, low quality reads, and clean reads, are shown. (TIF) (TIF) [file pone.0145532.s006.tif]

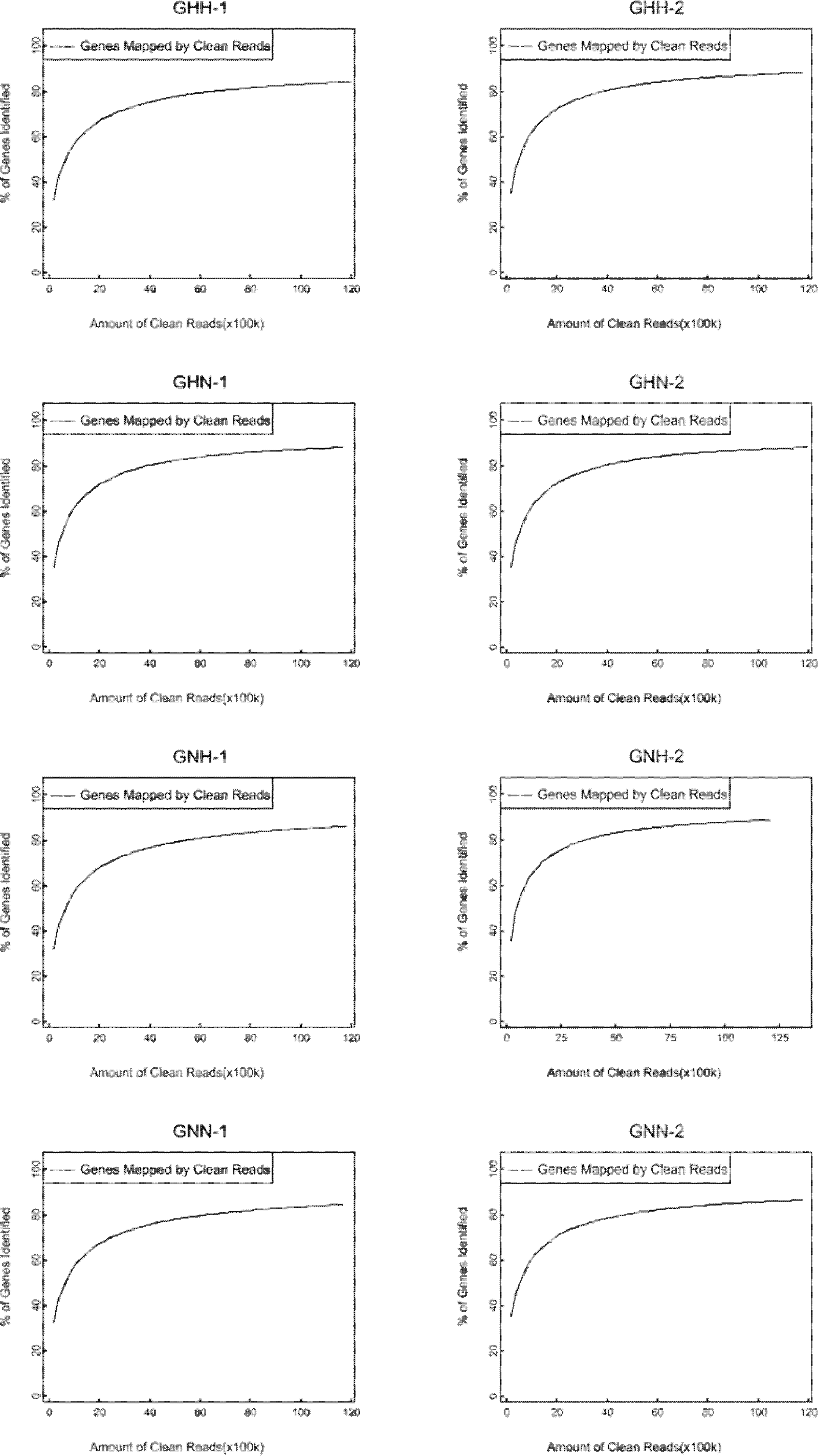

Supplement: S7 Fig — (TIF) [file pone.0145532.s007.tif]

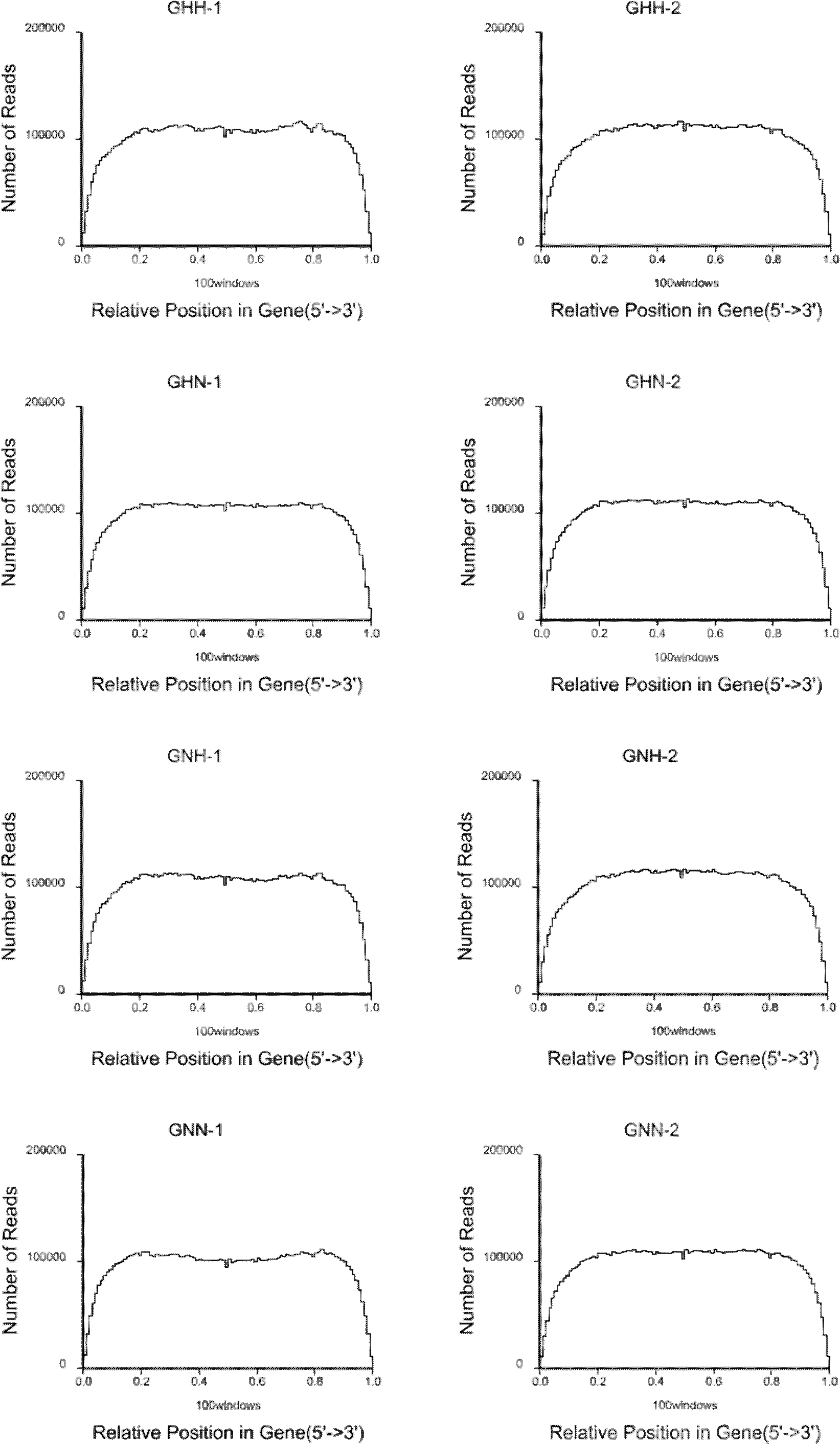

Supplement: S8 Fig — (TIF) [file pone.0145532.s008.tif]

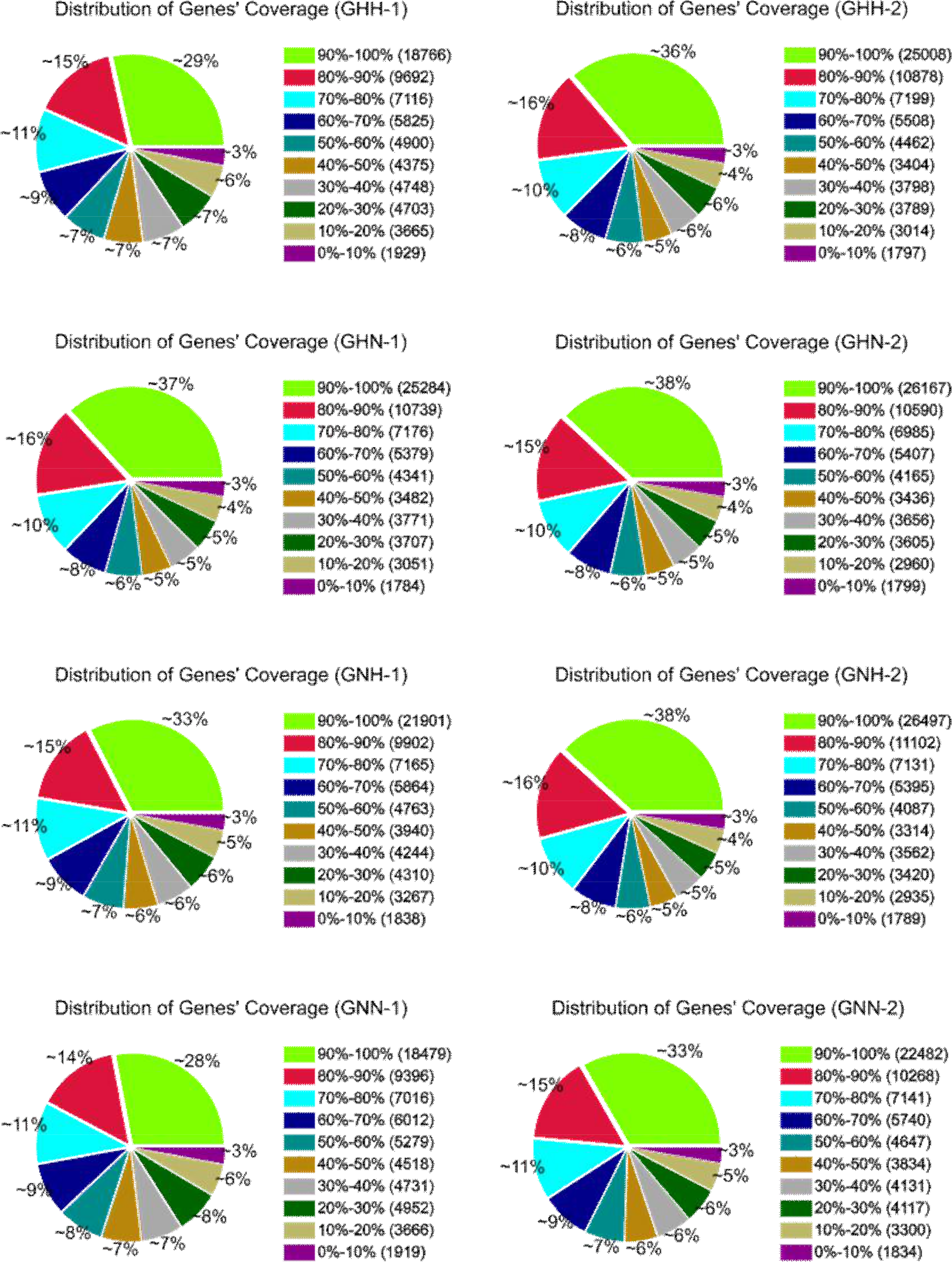

Supplement: S9 Fig — (TIF) [file pone.0145532.s009.tif]

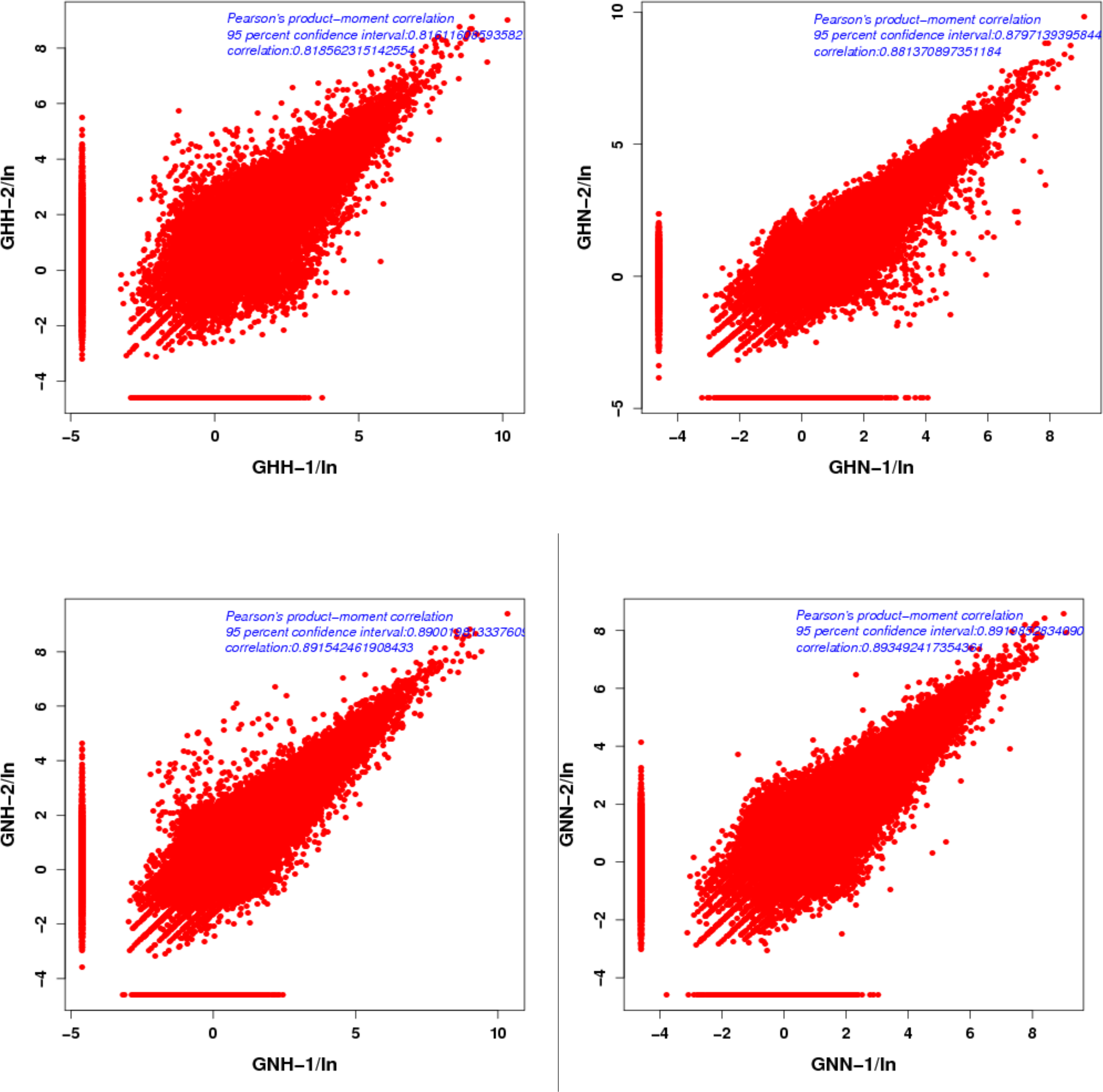

Supplement: S10 Fig — (TIF) [file pone.0145532.s010.tif]

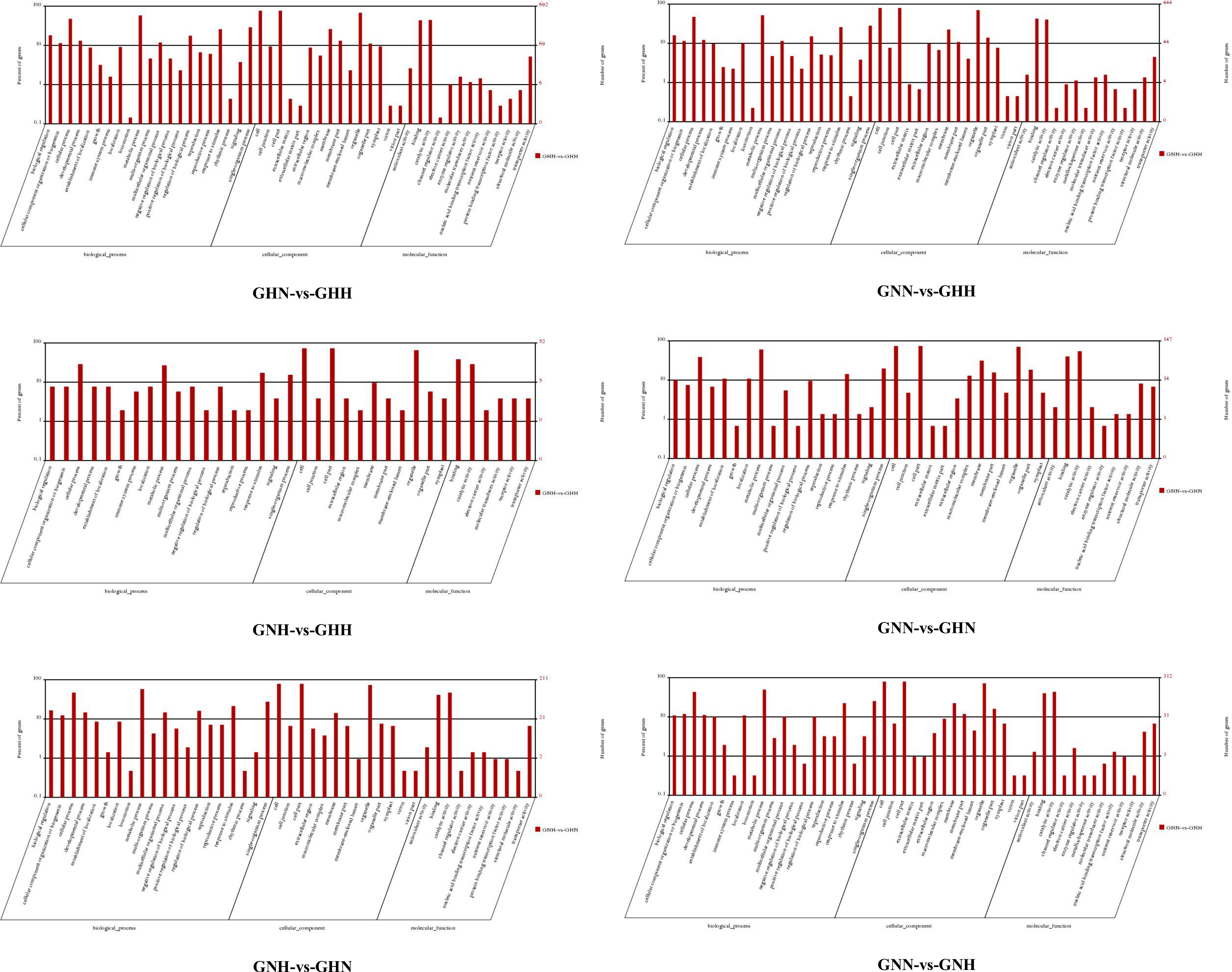

Supplement: S11 Fig — (TIF) [file pone.0145532.s011.tif]
